# Missed at birth: a cross-sectional analysis of national determinants and subnational changes in birth registration coverage in Kenya, using 2014 and 2022 Demographic and Health Survey (DHS) data

**DOI:** 10.1136/bmjph-2025-003850

**Published:** 2026-04-13

**Authors:** Bibian N Robert, Peter M Macharia, Viola Chepkurui, Joseph Kamau, Robert W Snow, Zhenlong Li, Emelda A Okiro

**Affiliations:** 1 Population & Health Impact Surveillance Group, KEMRI-Wellcome Trust Research Programme Nairobi, Nairobi, Nairobi County, Kenya; 2 Department of Geography, The Pennsylvania State University, University Park, 16802, Pennsylvania, USA; 3 Department of Public Health, Institute of Tropical Medicine, Antwerp, Belgium; 4 Department of Civil Registration Services (CRS), Nairobi, Nairobi, Kenya; 5 Centre for Tropical Medicine and Global Health, University of Oxford Nuffield Department of Medicine, Oxford, England, UK

**Keywords:** Public Health, Sociodemographic Factors, trends, Demography, Community Health

## Abstract

**Introduction:**

Registration of all births and deaths in sub-Saharan Africa remains inadequate. In Kenya, significant strides have been made, but progress has stalled in recent years. While some studies have examined factors influencing birth registration, national-level analytical assessments of these factors are limited. This study evaluates the progress and determinants of birth registration for children under 3 years in Kenya.

**Methods:**

We used cross-sectional data from the 2014 and 2022 Kenya Demographic and Health Surveys complemented by geospatial covariates of travel time and urbanicity. We computed the percentage of children (<3 years) registered with the civil authority at the national and subnational level (counties) and compared temporal changes against the United Nations (UN) ≥ 90% target. Binary and multivariable logistic regressions were used to assess determinants of birth registration in 2022, accounting for survey design.

**Results:**

National birth registration coverage improved from 69.0% (2014) to 76.0% (2022). Subnational coverage in 2014 ranged from 20.8% (West Pokot) to 96.4% (Nyeri) and from 46.4% (Marsabit) to 95.5% (Nyeri) in 2022. By 2022, only 17% (8/47) of counties met the UN’s ≥ 90% goal, with counties in the central region consistently performing better. Higher odds of birth registration were linked to health-facility births (adjusted OR (AOR)=2.44; 95% CI 2.07 to 2.89), immunisation (AOR=1.76; 95% CI 1.25 to 2.49), maternal age (35–39 years: AOR=1.61; 95% CI 1.22 to 2.12), higher education (AOR=1.39; 95% CI 1.03 to 1.89), media access (AOR=1.47; 95% CI 1.06 to 2.04), affiliation with the Catholic religion and the Kikuyu ethnic group. Households without bank accounts had lower odds (AOR=0.78; 95% CI 0.66 to 0.93). Notably, urbanicity and travel time to civil registration centres were not significantly associated with birth registration.

**Conclusion:**

National birth registration coverage has improved, but subnational disparities persist. Our findings show that in 2022, the health, education, financial and media sectors are associated with a higher likelihood of birth registration. These results underscore the need for the government and stakeholders to implement multisectoral strategies to strengthen Civil Registration and Vital Statistics and address socioeconomic and geographic inequalities critical to achieving universal birth registration coverage.

**WHAT IS ALREADY KNOWN ON THIS TOPIC**

- Birth registration remains low across sub-Saharan Africa, leaving millions of children unregistered. In Kenya, progress has stalled, with coverage declining in recent years.
- Existing analytical evidence on the determinants of birth registration in Kenya is limited to descriptive statistics, fragmented, focusing mainly on single subnational units. This narrow scope has obscured broader national trends and geographic disparities, particularly in relation to barriers such as travel time to civil registration centres.

**WHAT THIS STUDY ADDS**

- This study reveals significant subnational disparities in birth registration between 2014 and 2022, highlighting uneven progress toward universal coverage.
- It presents the first national-level analysis of factors influencing birth registration for children under age 3 in Kenya, identifying key socioeconomic and health service utilisation drivers.

**HOW THIS STUDY MIGHT AFFECT RESEARCH, PRACTICE OR POLICY**

- The findings underscore the need for multisectoral and locally tailored strategies to improve birth registration coverage and highlight the importance of addressing subnational challenges that are often obscured by national-level statistics.
- Highlights opportunities to better link civil registration with health systems to enhance birth registration while supporting child health monitoring, social protection and the fulfilment of children’s rights.

## Introduction

 The 2007 Lancet series, ‘Who Counts’, highlighted the inability of many countries to effectively record all births and deaths. This inadequacy was then labelled the ‘single most critical failure of development in the last 30 years’.^1^ Today, more than 15 years later, millions of birth events still go unregistered or are absent from national statistics, especially in Africa,^2^ which hampers global efforts and goals to ensure a legal identity for all by 2030 (Sustainable Development Goal (SDG) 16.9).^3^ Unregistered children who die before being recorded remain entirely unknown to the government, while those who survive face violations of their rights and miss out on essential services and interventions.

While most countries have passed laws that mandate the registration of births, ongoing issues mainly arise from incomplete Civil Registration and Vital Statistics (CRVS) systems, particularly in low- and lower-middle-income countries (LLMICs), affecting over 100 nations.^2 4^ A well-functioning CRVS system for birth registration can produce reliable birth statistics and has been associated with improved health outcomes.^5^ Accurate data are essential for monitoring global health, national and subnational development objectives and facilitating effective planning and implementation. This reduces dependence on sample household surveys, which are only conducted approximately every 5 years, or intricate estimation methods.^6^ For example, accurate data on live births are used to compute critical indicators including child mortality, maternal mortality^7 8^ and health service delivery, such as the proportion of births attended by skilled health personnel (SDG 3.1.2),^7^ and immunisation interventions, for example, in Rwanda.^9 10^

CRVS in numerous LLMICs requires individuals and families to actively seek out the registration of a birth at civil registration centres. This reliance on self-initiated actions instead of automated processes reflects the passive nature of CRVS and has worsened their limitations.^11 12^ To address this issue, several governments have introduced incentives designed to enhance birth registration.^13^ However, in addition to these systemic obstacles, individuals and communities face a range of cultural and socioeconomic issues that can hinder and/or delay the registration process.^14 15^ The primary barriers include limited awareness of registration requirements, bureaucratic challenges, inadequate registration infrastructure, staffing issues, high transportation costs and issues stemming from religious beliefs and social or ethnic discrimination.^16^

Complete birth registration occurs when every birth within a population is formally recorded in the CRVS system.^17^ Kenya has made significant strides in improving birth registration coverage driven by policy reforms such as decentralisation of registration services and increased public awareness campaigns. However, progress has largely plateaued in the recent years with marked declines in the national birth registration completeness since 2021.^17^,^19^ Persistent disparities across counties underscore the need for disaggregated county-level estimates and trend analysis to better understand and address these registration gaps.

Few studies in Kenya have interrogated factors influencing birth registration. While some regional and global studies have included Kenya,^20 21^ they often focus on comparisons with other countries, which obscures national specific determinants of registration. Additionally, subnational studies within Kenya have neglected certain areas, particularly in the western regions and the arid and semiarid northeastern parts.^22^,^26^ At the national level, the Kenya Civil Registration Services (CRS) highlights key trends in birth registration coverage by demographic characteristics, including gender, place of birth, mode of delivery, mother’s age, marital status and mother’s education level.^17 18^ However, there has been limited national analysis of the interaction of drivers of birth registration across all counties in Kenya.^27^

This study aims to examine the change in birth registration for children under 3 years between 2014 and 2022 and to identify the factors influencing birth registration in Kenya. The study uses data from the Kenya Demographic and Health Survey (KDHS) and geospatial covariates including travel time to civil registration offices (CROs), as a determinant of birth registration.

## Methods

### Country context

Following the enactment of the new constitution in 2010, Kenya adopted a devolved system of governance comprising 47 counties, which now serve as the primary administrative units for development planning and service delivery.^28^ The former eight provinces and the 47 county governments are shown in the online supplemental figure S1.

Registration of births in Kenya was introduced in 1904, using the colonial legal framework.^17 18^ The Births and Deaths Registration Act (CAP 149) was enacted in 1928,^29^ but nationwide implementation began in 1963. By 1971, all districts (now 47 counties) were declared compulsory registration areas with deputy registrars conducting household visits to register birth events.

Today, Kenya employs a community-based civil registration system.^18^ Assistant chiefs and medical personnel are the legal registration agents for births within the community and at health facilities, respectively. These agents record birth details in designated registers (Form B1) and issue birth notification slips to the informants or next of kin. The completed forms are sent to the subcounty civil registrar for verification and registration and then transmitted to the CRS statistics office by the 10th of every month. The national statistics department validates and compiles data into a master file for statistical analysis and production of reports such as the Kenya Vital Statistics Report.^17 18^

Births registered after 6 months are considered late registrations and are not counted in the national vital statistics data.^17^,^19^ To obtain a birth certificate, applicants must apply to the subcounty or national registry office, present the birth notification slips and pay a fee (approx. $0.47–$1.2) determined by the government. Generally, the CRS in Kenya is manual, with automation limited to urban counties like Nairobi and yet to be rolled out to other counties. A summary of the birth registration process is shown in online supplemental figure S2.

### Overview of methods

This is a cross-sectional study that uses data from the 2014 and 2022 KDHS. We estimated the weighted proportion of children under 3 whose births are registered with the civil authority. We then assessed changes in birth registration coverage across counties, grouped by the eight regions (former provinces) and benchmarked performances against the national averages and UN’s target of at least 90% coverage. To identify factors associated with birth registration, we restricted the analysis to the most recent survey (KDHS 2022) to ensure relevance to current policies and interventions. First, a univariate logistic regression was conducted followed by a multivariable logistic regression to assess adjusted associations.

### Data

The 2014 and 2022 KDHS are the two most recent nationally and country-representative surveys that include birth registration questions and align with the devolved governance system in Kenya. The KDHS household surveys employ a two-stage sampling design based on the Kenya Household Master Sample Frame.^30^,^32^ Detailed sampling procedures are documented in the KDHS reports.^3133^,^35^ Eligible respondents were women aged 15–49 years and men aged 15–54 years, who were usual residents of the sampled households or had slept there the night before the survey.^36^

### Participants

The analysis was restricted to children under 3 years of age who were usual residents of their households to minimise recall bias and because the majority of variables of interest in our study specifically targeted this age group.^37^ It also allowed for a clearer understanding of birth registration behaviours prior to the influence of school-related incentives such as the requirement of a birth certificate for school enrolment, which typically begins at age 5. A consistency check between the ages presented in months and years was conducted, and any misclassifications were corrected.

### Outcome variable: birth registration status

The outcome variable was birth registration status dichotomised as whether a child is registered with the civil authority or not at the time of the survey (online supplemental figure S2). A child was considered registered if the respondent (women aged 15–49) reported that a child’s birth was registered with the civil authority or if the child possessed a birth certificate, regardless of accompanying proof. Birth registration questions the response options for the 2014 and 2022 KDHS are summarised in online supplemental table S1).

### Independent variables

A review of the literature guided the understanding of the determinants of birth registration. The identified determinants^2226 38^,^62^ were broadly categorised as demand-side factors and supply-side factors (online supplemental figure S3). The demand-side factors were further reclassified as healthcare utilisation factors, child factors, parental factors and household factors. The supply-side factors were grouped into geographical and government/administrative factors. The conceptual framework summarising these determinants and their rationale is provided in online supplemental figure S3 and online supplemental table S2, respectively. The determinants were assembled from the KDHS 2022 based on their availability and completeness and/or extracted from modelled geospatial surfaces as summarised in online supplemental table S3.

#### Demand-side factors

These included determinants that influence the decision to register a child’s birth at an individual, household or community level. Specifically, child and parental demographic characteristics, socioeconomic status, cultural beliefs and awareness gained through education, interaction with healthcare personnel, media and services requiring birth certificates, such as opening a bank account (online supplemental figure S3).

#### Supply-side factors

Supply-side determinants influence the availability and accessibility of resources and services and include geographical aspects (urbanisation levels), elements inherent to the civil registration system (accessibility of registration locations or agents and governance) and administrative factors (political will and the legal framework). Urbanisation variables and travel times to CROs and health facilities were used as proxies for supply-side determinant factors.

Data on urbanisation continuum were extracted from the 2023 Global Human Settlement Raster Layer (GHSL) at 1 km × 1 km spatial resolution that incorporates satellite imagery and population data.^63^ The urban continuum was reclassified into urban areas (cities, large settlements, dense and semidense towns and suburbs) and rural areas (villages, dispersed areas and very low-density areas).^63 64^ Estimates of travel time to health facilities were available from Moturi *et al* ^65^ and had been generated using a least cost path algorithm.^66^ We used the same approach to generate estimates of travel time surface to CROs (online supplemental figure S5, online supplemental table S4 and S5). Mean travel times to health facilities and CROs were extracted for each Demographic and Health Survey (DHS) cluster. To account for DHS cluster displacement, we extracted values using buffers of 5 km for rural clusters and 2 km for urban clusters.^67^ Urbanisation class for each buffer was then assigned using the majority classification based on the GHSL layer.

#### Exclusion criteria and missingness

Only children with complete data across all determinants were included in the analysis, regardless of their birth registration status (online supplemental figure S4). Exclusion criteria included deceased children, children not listed in a household due to not living with their biological parents or where a woman was ineligible for the interview (eg, aged outside the 15–49 years). DHS determinants that were restricted to specific subsets of children (eg, antenatal care, which applied to last born only) were excluded, and other proxies such as skilled birth attendance, place of birth and immunisation applicable to all children under 3 were used instead. Data completeness was high across the selected variables (ranging from 99.9% to 100%) with only minimal missingness. ‘Don't know’ responses were absent across all included variables except maternal occupation (0.08%) and were excluded from the analysis.

### Analysis

#### Birth registration coverage 2014 versus 2022

To assess the progress (or lack thereof) in birth registration coverage between 2014 and 2022, we computed the weighted proportions of children under 3 whose births were registered nationally and in each county. Birth registration coverage refers to the percentage of children under 3 years old whose births have been officially recorded with a civil authority. ‘Yes’ responses formed the numerators, and all children under 3 years formed the denominator. The relative change in birth registration coverage for each county was evaluated.

We also compared the county registration rates with the national average for each survey year, and with the UN’s target of at least 90% birth registration coverage, used as a minimum progress threshold towards universal birth registration by 2030 (SDG Target 16.9).

### Statistical analysis of determinants of birth registration

A univariate logistic regression model was fit to examine the bivariate relationship between birth registration status (registered=1 vs not registered=0) and each independent variable *x_i_* (equWL1). Next, a multivariable logistic regression was fit, and the best model that best explains the variability in birth registration was selected based on the lowest Akaike Information Criterion using backward stepwise regression. Collinearity diagnostics were assessed using the variance inflation factors (VIF), with a cut-off of 10 (the standard practice).^68^

All analyses accounted for the DHS complex survey design using the survey package in R (V.4.4–2), incorporating stratification, clustering and sampling weights. A complete-case analysis approach was used, as described in the missingness section, and no sensitivity analyses were performed.

EquWL1. General form of logistic regression accounting for complex survey design.


$$\log(\frac{\pi_{hjik}}{1-\pi_{hjik}})=\beta_{0}+\beta_{1}x_{1,hjik}+\beta_{2}x_{2,hjik}+\ldots +\beta_{p}x_{p,hjik}$$

where *hjik* refers to the hierarchical structure of survey design:

h=the stratum (urban or rural); j=the clusters which are the primary sampling units (PSU); i=households, the secondary sampling unit (SSU) within each PSU; k=individuals within the SSU.

## Results

### Descriptive results

**Figure 1:**
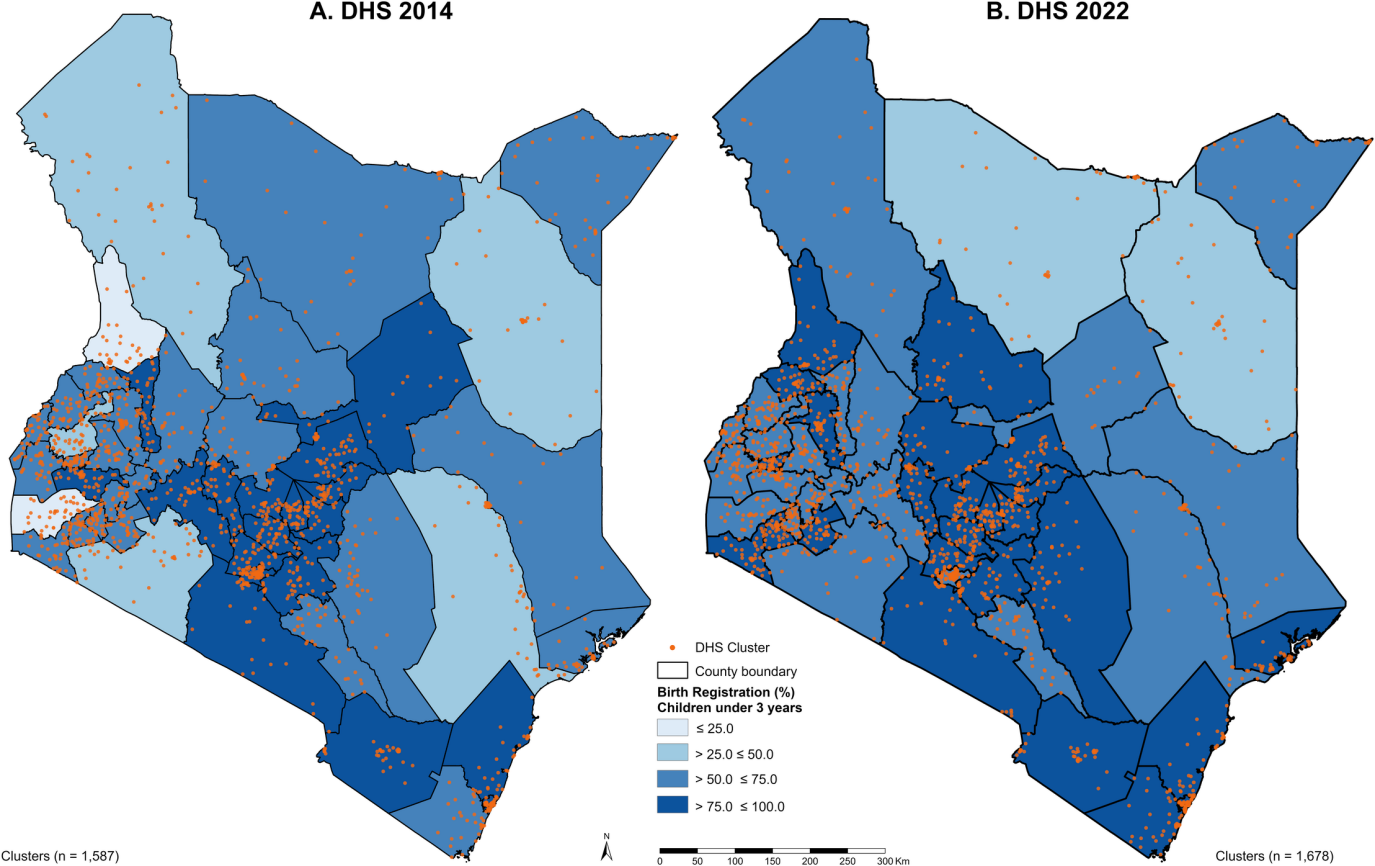
Spatial distribution of included clusters and birth registration coverage for children under 3 years: (A) DHS 2014 and (B) DHS 2022. DHS, Demographic and Health Survey.

A total of 11 662 children under 3 years of age across 1587 clusters were included in the coverage analysis for 2014 and 10 491 children across 1678 clusters for 2022. The spatial distribution of these clusters and birth registration coverage for children under 3 across counties in both years is shown in figure 1. For 2022, a subset of 9131 children under 3 with complete dataset across all selected independent variables was included in the analysis of determinants of birth registration (online supplemental figure S4).

Table 1 summarises the birth registration status by the selected independent variables. The registration rate was higher among older children, those who had contact with health services (immunised, delivered in health facilities and delivered by skilled birth attendants) and children whose mothers had secondary education, employed formally and had weekly media exposure. Registration rates also varied significantly (p<0.05) across ethnic groups, religion, wealth quintiles, access to media devices and bank account ownership. Children with low registration rates included those residing in rural areas and at longer travel times to health facilities and CROs. No significant differences in registration were observed by children’s gender, mothers’ experience of child death, marital status and sex of household head.

**Table 1:** Descriptive summary of birth registration status for children under 3 years, by the selected independent variables.

Variable	Birth registration status (<3 years)				P value†
	No, weighted, n=2194 (24%)*	95% CI 23%, 25%	Yes, weighted, n=6937 (76%)*	95% CI 75%, 77%	
Child factors					
Age					0.018
<1 years	866 (26.1%)	24%, 28%	2456 (73.9%)	72%, 76%	
1–2 years	702 (23.4%)	21%, 25%	2298 (76.6%)	75%, 79%	
2–3 yrs	626 (22.3%)	20%, 24%	2184 (77.7%)	76%, 80%	
Sex					0.6
Male	1105 (23.7%)	22%, 26%	3556 (76.3%)	74%, 78%	
Female	1089 (24.4%)	23%, 26%	3382 (75.6%)	74%, 77%	
Birth order					<0.001
1	621 (24.1%)	22%, 26%	1955 (75.9%)	74%, 78%	
2	434 (20.0%)	18%, 22%	1739 (80.0%)	78%, 82%	
3	348 (21.3%)	19%, 24%	1290 (78.7%)	76%, 81%	
4+	790 (28.8%)	27%, 31%	1953 (71.2%)	69%, 73%	
Birth interval					<0.001
0 (first born)	627 (24.0%)	22%, 26%	1986 (76.0%)	74%, 78%	
8–23	300 (28.8%)	25%, 32%	744 (71.2%)	68%, 75%	
24–47	733 (28.2%)	26%, 31%	1865 (71.8%)	69%, 74%	
48+	534 (18.6%)	17%, 21%	2342 (81.4%)	79%, 83%	
Healthcare utilisation indicators					
Birth attendant					<0.001
None	61 (46.4%)	38%, 55%	71 (53.6%)	45%, 62%	
Skilled	1666 (20.9%)	20%, 22%	6300 (79.1%)	78%, 80%	
Unskilled	466 (45.2%)	42%, 49%	566 (54.8%)	51%, 58%	
Birth place					<0.001
Home/other	565 (49.0%)	46%, 52%	588 (51.0%)	48%, 54%	
Health facility	1629 (20.4%)	19%, 22%	6349 (79.6%)	78%, 81%	
Immunisation status					<0.001
Not immunised	116 (46.8%)	41%, 53%	132 (53.2%)	47%, 59%	
Partly immunised	1127 (26.0%)	24%, 28%	3202 (74.0%)	72%, 76%	
Fully immunised	951 (20.9%)	19%, 22%	3603 (79.1%)	78%, 81%	
Maternal factors					
Mother’s age					<0.001
15–24	838 (28.4%)	26%, 31%	2111 (71.6%)	69%, 74%	
25–29	594 (22.3%)	20%, 25%	2067 (77.7%)	75%, 80%	
30–34	403 (21.9%)	19%, 25%	1435 (78.1%)	75%, 81%	
35–39	245 (20.7%)	18%, 24%	938 (79.3%)	76%, 82%	
40–49	114 (22.8%)	18%, 28%	386 (77.2%)	72%, 82%	
Child loss					0.4
No	1965 (23.9%)	23%, 25%	6268 (76.1%)	75%, 77%	
Yes	229 (25.5%)	22%, 29%	670 (74.5%)	71%, 78%	
Parity					<0.001
1	564 (24.5%)	22%, 27%	1732 (75.5%)	73%, 78%	
2	458 (19.9%)	18%, 22%	1842 (80.1%)	78%, 82%	
3	352 (21.1%)	19%, 24%	1319 (78.9%)	76%, 81%	
4+	821 (28.7%)	26%, 31%	2044 (71.3%)	69%, 74%	
Marital status					0.086
Single	251 (27.1%)	24%, 31%	677 (72.9%)	69%, 76%	
Married	1758 (23.4%)	22%, 25%	5746 (76.6%)	75%, 78%	
Formerly married	185 (26.5%)	22%, 31%	514 (73.5%)	69%, 78%	
Maternal education					<0.001
No education	395 (41.7%)	38%, 45%	554 (58.3%)	55%, 62%	
Primary	921 (27.6%)	25%, 30%	2415 (72.4%)	70%, 75%	
Secondary/higher	878 (18.1%)	17%, 20%	3969 (81.9%)	80%, 83%	
Maternal occupation					<0.001
Not working	1127 (27.2%)	25%, 29%	3024 (72.8%)	71%, 75%	
Formal	198 (14.5%)	12%, 17%	1163 (85.5%)	83%, 88%	
Manual	182 (24.6%)	21%, 29%	555 (75.4%)	71%, 79%	
Others	687 (23.8%)	22%, 26%	2195 (76.2%)	74%, 78%	
Media access					<0.001
Not at all	766 (33.2%)	31%, 36%	1540 (66.8%)	64%, 69%	
At least once a week	1428 (20.9%)	19%, 23%	5397 (79.1%)	77%, 81%	
Household factors					
Household head					0.9
Male	1582 (24.0%)	22%, 26%	5014 (76.0%)	74%, 78%	
Female	613 (24.2%)	22%, 26%	1923 (75.8%)	74%, 78%	
Bank account					<0.001
No	1489 (29.6%)	28%, 31%	3544 (70.4%)	69%, 72%	
Yes	705 (17.2%)	16%, 19%	3393 (82.8%)	81%, 84%	
Media devices					<0.001
None	145 (42.6%)	36%, 49%	195 (57.4%)	51%, 64%	
At least one	1454 (27.8%)	26%, 30%	3785 (72.2%)	70%, 74%	
All three	595 (16.8%)	15%, 19%	2957 (83.2%)	81%, 85%	
Wealth index					<0.001
Poorest	802 (37.0%)	34%, 40%	1364 (63.0%)	60%, 66%	
Poorer	447 (26.5%)	24%, 29%	1243 (73.5%)	71%, 76%	
Middle	345 (21.5%)	19%, 24%	1260 (78.5%)	76%, 81%	
Richer	357 (19.2%)	17%, 22%	1500 (80.8%)	78%, 83%	
Richest	243 (13.4%)	10%, 17%	1570 (86.6%)	83%, 90%	
Religion					<0.001
Catholic	332 (20.1%)	17%, 23%	1318 (79.9%)	77%, 83%	
Protestant/other Christian	1484 (24.0%)	22%, 26%	4712 (76.0%)	74%, 78%	
Islam	291 (32.4%)	28%, 37%	607 (67.6%)	63%, 72%	
No religion	30 (26.1%)	17%, 37%	86 (73.9%)	63%, 83%	
Others	56 (20.7%)	15%, 28%	214 (79.3%)	72%, 85%	
Ethnicity					<0.001
Embu	16 (18.3%)	10%, 31%	73 (81.7%)	69%, 90%	
Kalenjin	425 (30.2%)	27%, 34%	980 (69.8%)	66%, 73%	
Kamba	142 (17.1%)	13%, 21%	691 (82.9%)	79%, 87%	
Kikuyu	167 (12.2%)	9.6%, 15%	1204 (87.8%)	85%, 90%	
Kisii	93 (20.0%)	15%, 27%	374 (80.0%)	73%, 85%	
Luhya	362 (25.7%)	22%, 30%	1045 (74.3%)	70%, 78%	
Luo	278 (27.5%)	24%, 31%	733 (72.5%)	69%, 76%	
Maasai	127 (37.0%)	30%, 45%	217 (63.0%)	55%, 70%	
Meru	47 (11.5%)	7.5%, 17%	362 (88.5%)	83%, 93%	
Mijikenda	106 (20.5%)	17%, 25%	414 (79.5%)	75%, 83%	
Somali	168 (39.0%)	31%, 47%	264 (61.0%)	53%, 69%	
Taita/Taveta	2 (4.3%)	1.8%, 9.6%	48 (95.7%)	90%, 98%	
Others	259 (32.7%)	28%, 38%	532 (67.3%)	62%, 72%	
Geographical factors					
Urbanicity					<0.001
Rural	868 (29.0%)	27%, 31%	2124 (71.0%)	69%, 73%	
Urban	1326 (21.6%)	20%, 23%	4813 (78.4%)	77%, 80%	
Travel time CROs (hours)	1.42	1.3, 1.5	1.05	1.0, 1.1	<0.001
Travel health facilities (hours)	0.31	0.27, 0.34	0.21	0.19, 0.22	<0.001

* n (%); (mean)

† 2 test with Rao and Scott’s second-order correction; Wilcoxon rank-sum test for complex survey samples

CRO, civil registration offices.

### Coverage of birth registration 2014 versus 2022

**Figure 2:**
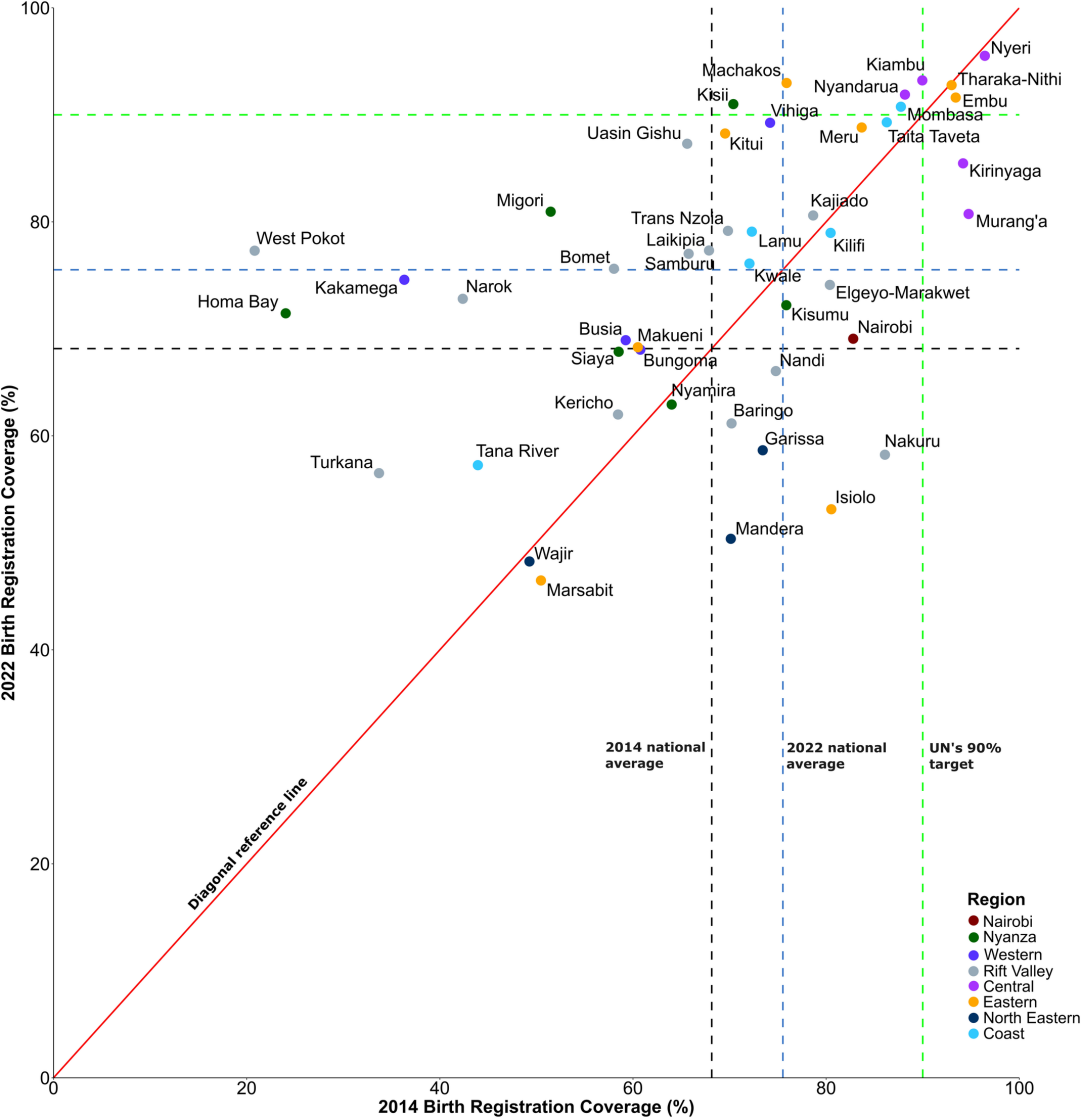
County-level comparison of birth registration coverage in 2014 and 2022, against the national average and the UN’s 90% target. The counties are colour-coded by the regions. The dashed black, blue, and green lines indicate the 2014 national coverage, 2022 national coverage, and the UN’s ≥ 90% target, respectively.

Nationally, the coverage of birth registration rose from 69.0% in 2014 to 76.0% in 2022. At the county level, coverage varied in 2014, with West Pokot at 20.8% and Nyeri at 96.4% (figures 1A and 2). In 2022, coverage ranged from 46.4% in Marsabit to 95.5% in Nyeri (figures 1B and 2). More than half of the counties (29 out of 47) demonstrated improvement over the two time periods, as illustrated in figures2  3. Notably, counties such as West Pokot, Homa Bay and Kakamega more than doubled their coverage (figure 2). In contrast, a subset of counties, including Isiolo, Nakuru, Mandera and Garissa, experienced the largest declines, each exceeding 20%. Among the urban counties (Nairobi and Mombasa), only Mombasa saw an increase, reaching the UN’s ≥ 90% target in 2022. Similarly, Machakos, Nyandarua and Kisii also improved and met the UN’s target in 2022. Counties like Nyeri, Embu and Tharaka-Nithi sustained coverage above the UN’s target, even with minor declines. By 2022, only eight counties met the UN’s ≥ 90% goal, an increase from 6 in 2014. Meanwhile, the count of counties falling below the national average rose from 8 in 2014 to 17 by 2022.

**Figure 3:**
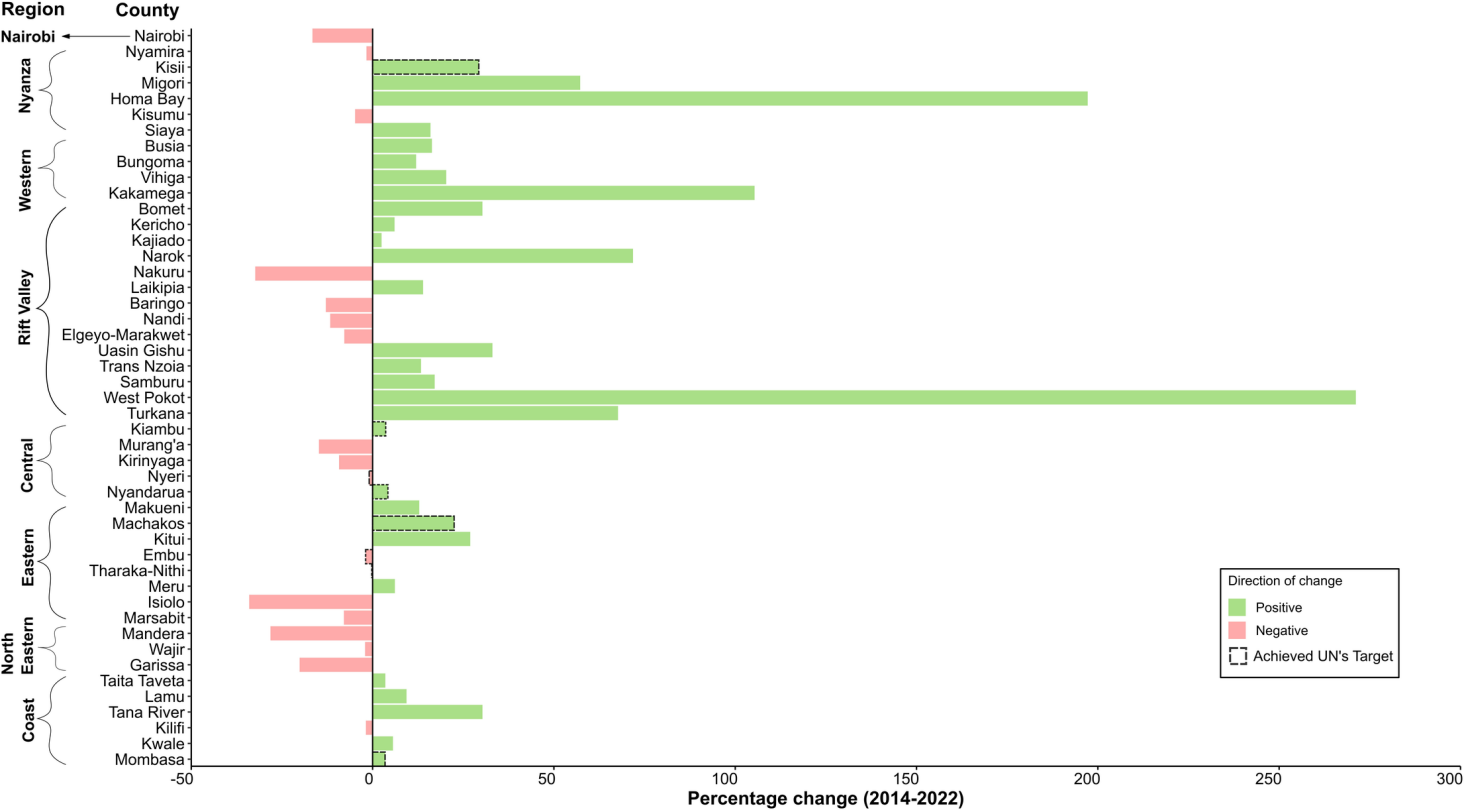
Relative change in birth registration coverage for children under 3 between 2014 and 2022, highlighting counties that achieved the 90% UN’s birth registration target.

Regional patterns indicate that counties in the central region, including Kiambu, Murang’a, Kirinyaga, Nyeri and Nyandarua, consistently had birth registration coverage above the national average (figure 2). In contrast, counties in the North-eastern region recorded persistently lower rates, with all counties in this area experiencing declines in 2022. Additionally, disparities within regions were noticeable, as counties such as Marsabit and Isiolo in the eastern region, along with Tana River in the coastal region, had lower registration rates compared with their peers. Meanwhile, Vihiga reported significantly higher rates than other counties in Nyanza.

### Univariate and multivariable logistic regression results

Table 2 shows results of running the univariate and multivariable logistic regression on factors associated with birth registration. After the backwards step-regression process, 15 out of 23 variables were retained in the final multivariable model. Multicollinearity was not a concern as factors in the final multivariable model had low VIF (<2) values.

**Table 2:** Determinants of birth registration for children under 3 in 2022.

Variable	Univariate		Multivariable	
	OR (95% CI)	P value	AOR (95% CI)	P value
Child factors				
Age				
<1 years	—		—	
1 year	1.15 (0.99, 1.34)	0.064	1.10 (0.90, 1.34)	0.35
2 years	1.23 (1.07, 1.42)**	0.004	1.15 (0.95, 1.39)	0.14
Sex				
Male	—			
Female	0.97 (0.85, 1.09)	0.57		
Birth order				
1	—			
2	1.27 (1.07, 1.51)**	0.006		
3	1.18 (0.97, 1.42)	0.093		
4+	0.78 (0.67, 0.92)**	0.002		
Birth interval				
0	—		—	
8–23	0.78 (0.64, 0.95)*	0.012	0.92 (0.60, 1.40)	0.69
24–47	0.80 (0.69, 0.94)**	0.005	0.86 (0.56, 1.32)	0.49
48+	1.38 (1.16, 1.65)***	<0.001	1.01 (0.64, 1.60)	0.96
Healthcare utilisation indicators				
Birth attendant				
None	—			
Skilled	3.27 (2.28, 4.70)***	<0.001		
Unskilled	1.05 (0.72, 1.54)	0.80		
Birth place				
Home/other	—		—	
Health facility	3.75 (3.23, 4.35)***	<0.001	2.44 (2.07, 2.89)***	<0.001
Immunisation status				
Not immunised	—		—	
Partly immunised	2.50 (1.92, 3.26)***	<0.001	1.51 (1.07, 2.12)*	0.018
Fully immunised	3.33 (2.56, 4.34)***	<0.001	1.76 (1.25, 2.49)**	0.001
Maternal factors				
Mother’s age				
15–24	—		—	
25–29	1.38 (1.17, 1.63)***	<0.001	1.27 (1.03, 1.57)*	0.027
30–34	1.41 (1.18, 1.69)***	<0.001	1.39 (1.06, 1.82)*	0.017
35–39	1.52 (1.26, 1.83)***	<0.001	1.61 (1.22, 2.12)***	<0.001
40–49	1.34 (0.99, 1.83)	0.060	1.44 (0.95, 2.18)	0.082
**Child loss**				
Yes	—			
No	1.09 (0.90, 1.33)	0.37		
Parity				
1	—		—	
2	1.31 (1.08, 1.59)**	0.007	1.35 (0.86, 2.13)	0.20
3	1.22 (1.00, 1.48)*	0.048	1.20 (0.74, 1.93)	0.46
4+	0.81 (0.69, 0.95)*	0.011	1.14 (0.68, 1.91)	0.61
Marital status				
Single	—			
Married	1.21 (1.00, 1.48)	0.055		
Formerly married	1.03 (0.77, 1.39)	0.84		
Maternal education				
No education	—		—	
Primary	1.87 (1.56, 2.25)***	<0.001	1.08 (0.81, 1.43)	0.61
Secondary/higher	3.23 (2.68, 3.89)***	<0.001	1.39 (1.03, 1.89)*	0.034
Maternal occupation				
Not working	—		—	
Formal	2.20 (1.77, 2.73)***	<0.001	1.26 (1.00, 1.59)	0.053
Manual	1.14 (0.88, 1.47)	0.32	0.90 (0.68, 1.19)	0.48
Others	1.19 (1.02, 1.39)*	0.027	0.91 (0.77, 1.09)	0.31
Media access†				
Not at all	—		—	
At least once a week	1.88 (1.63, 2.17)***	<0.001	1.15 (0.96, 1.37)	0.13
Household factors				
Household head				
Male	—			
Female	0.99 (0.86, 1.14)	0.89		
Bank account				
Yes	—		—	
No	0.49 (0.43, 0.57)***	<0.001	0.78 (0.66, 0.93)**	0.005
Media devices‡				
None	—		—	
At least one	1.93 (1.46, 2.55)***	<0.001	1.27 (0.97, 1.67)	0.087
All three	3.69 (2.74, 4.97)***	<0.001	1.47 (1.06, 2.04)*	0.020
Wealth index§				
Poorest	—			
Poorer	1.63 (1.39, 1.92)***	<0.001		
Middle	2.15 (1.79, 2.58)***	<0.001		
Richer	2.47 (1.99, 3.06)***	<0.001		
Richest	3.80 (2.82, 5.13)***	<0.001		
Religion				
Catholic	—		—	
Protestant/other Christian	0.80 (0.65, 0.99)*	0.036	0.78 (0.63, 0.97)*	0.024
Islam	0.53 (0.39, 0.70)***	<0.001	0.76 (0.50, 1.16)	0.21
No religion	0.71 (0.42, 1.22)	0.22	0.96 (0.57, 1.61)	0.88
Others	0.97 (0.61, 1.53)	0.88	1.08 (0.69, 1.70)	0.74
Ethnicity				
Kikuyu	—		—	
Embu	0.62 (0.29, 1.33)	0.22	0.64 (0.31, 1.33)	0.23
Kalenjin	0.32 (0.23, 0.44)***	<0.001	0.50 (0.36, 0.70)***	<0.001
Kamba	0.67 (0.46, 0.99)*	0.047	0.84 (0.56, 1.27)	0.41
Kisii	0.56 (0.34, 0.90)*	0.016	0.63 (0.38, 1.04)	0.072
Luhya	0.40 (0.29, 0.56)***	<0.001	0.51 (0.37, 0.71)***	<0.001
Luo	0.37 (0.27, 0.50)***	<0.001	0.45 (0.33, 0.63)***	<0.001
Maasai	0.24 (0.15, 0.36)***	<0.001	0.56 (0.36, 0.88)*	0.011
Meru	1.07 (0.62, 1.84)	0.81	1.34 (0.77, 2.32)	0.30
Mijikenda	0.54 (0.38, 0.77)***	<0.001	0.95 (0.64, 1.40)	0.79
Somali	0.22 (0.14, 0.33)***	<0.001	0.64 (0.35, 1.16)	0.14
Taita/Taveta	3.10 (1.25, 7.68)*	0.015	4.16 (1.65, 10.5)**	0.003
Others	0.29 (0.20, 0.40)***	<0.001	0.60 (0.41, 0.89)*	0.011
Geographical factors				
Urbanicity				
Urban	—			
Rural	0.67 (0.58, 0.79)***	<0.001		
Travel time health facilities (hours)	0.47 (0.35, 0.62)***	<0.001	1.18 (0.90, 1.55)	0.23
Travel time CROs (hours)	0.78 (0.74, 0.83)***	<0.001	0.94 (0.87, 1.01)	0.072

* p<0.05; **p<0.01; ***p<0.001.

† Refers to how frequently a mother consumes information from the media.

‡ Refers to whether a household owns a media device (TV, radio or smartphone) sometimes family members can relay information on birth registration.

§ A composite measure of the cumulative living standard of a household, computed using the principal component analysis (PCA) using data on household’s ownership of selected set of assets, such as televisions, bicycles and cars; dwelling characteristics such as flooring material, etc. Households are divided into five wealth quintiles (poorest, poor, medium, richer, richest) based on their wealth scores.^97^

Child-level factors including age and birth interval were not associated with birth registration. Children born in health facilities and those immunised had higher odds of birth registration than those born at home or not immunised. Among maternal factors, the odds of birth registration increased with age, with the strongest likelihood observed in mothers aged 35–39 (AOR = 1.61; 95% CI 1.22 to 2.12; p < 0.001). However, the association among mothers aged 40–49 was not statistically significant (p=0.082). Mothers with secondary or higher education levels were 3.23 times more likely to register their children than those without education (95% CI 1.03 to 1.89, p=0.034).

Households without bank account holders had 22% lower odds of registering a child’s birth than those with bank accounts (AOR=0.78, 95% CI 0.66 to 0.93, p=0.005). Additionally, those equipped with all three media types (TV, radio and phone) were 1.47 times more likely to register their children than those lacking any media device (95% CI 1.06 to 2.04). Most tribes in Kenya exhibited reduced odds of birth registration relative to the Kikuyu, the largest tribe in the country. Further, households practising Protestant or other Christian faiths had statistically significantly lower odds (AOR=0.78, 95% CI 0.63, 0.97, p value=0.024) of registering a child’s birth than Catholic households.

None of the geographical factors, including urbanicity and travel time to health facilities and CROs, nor maternal characteristics such as parity, occupation or weekly media access, were significantly associated with the odds of birth registration.

## Discussion

Kenya has made notable progress in improving birth registration coverage over the years, but achieving universal coverage has been challenging, with progress stagnating and/or reducing in recent years.^19^ This study had two main objectives. First, using KDHS data, we examined changes in birth registration coverage for children under 3 years, revealing an improvement nationally from 69.9% in 2014 to 76.0% in 2022, alongside persistent subnational disparities. Second, using the most recent KDHS 2022 data and modelled geospatial data, we assessed the combined influence of selected determinants of birth registration in Kenya. The results indicate that hospital delivery, child immunisation, mother’s age (35–39 years), higher maternal education, bank account ownership, access to multiple media devices and Catholic religion were associated with higher odds of birth registration.

The rise in birth registration coverage reflects efforts that have been made to enhance birth registration coverage in Kenya.^69^,^73^ Notably, the number of CROs expanded from 69 in 2003 to 107 by 2012^74^ to about 167 CROs in 2025, including ongoing efforts to extend services to all subcounties and constituencies.^75^ Despite such initiatives, significant subnational disparities in progress persist, with the number of counties below the national average doubling by 2022. According to UNICEF, birth registration coverage in SSA is approximately 51%, while in East Africa averages around 41%.^76^ While Kenya’s coverage is above these regional and subregional averages, the persistent internal disparities highlight unique county-specific dynamics shaping registration trajectories. Localised interventions such as increased investment in Maternal, Newborn and Child Health (MNCH) in Kakamega county,^77 78^ improving data collection systems for CRVS systems and training CRS staff in counties including Kakamega, Homa Bay, Machakos, Siaya and Migori^79^ positively impacted birth registration. Other most improved counties like West Pokot benefited from a 30-day rapid results initiative to clear all unregistered births and backlog in the issuance of birth.^80^ While urban counties like Mombasa improved, Nairobi experienced a drop in coverage, reflecting unique urban-specific challenges that need further investigation.^81^

Regional disparities are also evident where all counties in the Western region improved while those in the northeastern region, often marginalised, saw a drop. Counties in the central region, known for maintaining high birth registration rates above the national average, serve as role models for others. Nevertheless, the declines observed from 2014 to 2022 in these typically high-performing counties highlight the need to focus on the sustainability of CRVS systems along with efforts to enhance performance in counties with lower registration rates. The COVID-19 pandemic exposed the weaknesses of manual CRVS systems that depend on in-person interactions, as registration of vital events faced significant disruptions. This underlines the necessity of fortifying these systems. 82,84These insights suggest that while national-level interventions are beneficial in propelling progress, addressing regional and country-specific challenges is crucial to maximise impact.

The determinant analysis shows that at the national level, utilisation of healthcare services is associated with higher odds of birth registration, corroborating findings across multiple countries.^41^,^44^ This universal finding underscores the synergy between MNCH services and birth registration coverage. In Kenya, healthcare personnel initiate the birth registration process by notifying the CRS of events as they occur. However, challenges such as a shortage of notification forms and delays in submitting the filled forms to the CRS can hinder timely registration.^85 86^ Though geographical access to health facilities did not show significant associations with birth registration, the health sector, through the Ministry of Health, has several avenues to enhance birth registration. Healthcare personnel could promote birth registration facilities while interacting with parents seeking care, and community health workers could raise awareness within communities.^87^ Introducing integrated digital CRVS systems, like those in Rwanda, along with automated birth notifications and the issuance of birth certificates in health facilities, can enhance efficiency.

Older women and those with higher education levels were more inclined to register their children, similar to other studies.^39 43 46 52^ This may reflect prior experience with the birth registration process^51^ and awareness of its benefits through interactions with formal education system or exposure such as travel. Media exposure, particularly access to TV, radio and smartphones, remains crucial to improving birth registration. Kenyan households have access to all the top media channels—TV, radio and smartphones—and are presented with multiple channels through which information on birth registration can be relayed, increasing the odds of birth registration. Smartphones complement the other channels by providing easy access for many citizens where registration can be initiated through government portals such as the eCitizen.^88^

Ethnic diversity also influenced birth registration.^89^ Some ethnic groups may face unique challenges or have differing perceptions of its importance, which can either facilitate or hinder birth registration.^22 50 60^ Communities in central Kenya, predominantly inhabited by the Kikuyus, have historically shown high birth registration coverages, with a nearly universal understanding of its benefits and utilisation of MNCH services.^26^ In contrast, marginalised regions like northern Kenya, home to border tribes like the Somali, have faced unique barriers. These include fewer registration offices,^90^ low coverages in antenatal and health facility deliveries^33^ and, until recently, rigorous background checks before receiving birth certificates due to national security concerns.^91^ The influence of religion on birth registration was evident, likely driven by religious beliefs and practices that shape women’s reproductive health behaviours such as hospital deliveries.^57^ In some settings like Ethiopia, religious baptismal cards may be mistaken for birth certificates, hindering official birth registration. However, Kenya recognises such documents as valid for birth registration, potentially increasing the chances of registration among certain faiths, such as Catholics that issue baptismal cards.^92 93^

Notably, marital status was not a significant determinant of birth registration, likely reflecting sensitisation campaigns led by CRS, philanthropic agencies and CHVs which clarified that a marriage certificate was not required for birth registration.^94^ Previously, such misconceptions discouraged single mothers. Unlike countries like Indonesia, Kenya does not require marital documents, but only a birth notification form from a health facility or the assistant chief’s office for home births.^95^

Geographical access to birth registration sites has been identified as a significant barrier to birth registration.^38 39^ However, this factor is often omitted from analyses, or proxies for remoteness of registration are used instead.^40^ Even when spatial access is incorporated in analysis, distances are broadly categorised (eg, under and over 10 km),^25^ or fail to account for transportation methods, travel speeds and transport obstacles.^96^ It is generally expected that greater distances (longer travel times) to CROs, particularly for rural residents, would raise financial and opportunity costs (eg, transport) and impede birth registration.^23 25 26 38^ Our study found no significant association between travel distance to CROs and birth registration, consistent with a local survey conducted in Kwale (coastal Kenya) that used reported travel time estimates.^23^ The requirement for all young children in Kenya to present a birth certificate on school enrolment may have raised general awareness among parents, motivating them to overcome geographical barriers to access birth registration services.^46 47^ Nonetheless, such incentives fail to combat late birth registration (after 6 months of birth). Qualitative studies in some Kenyan regions reported distance to the CROs as a key challenge.^23 25 26^ This underscores the need to examine these relationships locally as unique patterns may be obscured in national-level analyses. Such analyses can reveal localised significant relationships, even for variables like parity, household head and urbanicity, that were non-significant in this study.

### Limitations

Though this study provides the first country-level insights into Kenya’s birth registration landscape, it has several limitations. The KDHS survey relied on self-reported information on birth registration without requiring proof such as birth certificates, which may introduce reporting biases. The analysis included only children born within 3 years preceding the survey with complete data across the selected variables. This approach minimised recall bias and prevented the exclusion of many children with missing variables, such as on marriage certificate ownership that targeted only married women. However, we acknowledge that this approach may introduce selection bias and limit generalisability if recent births differ systematically from earlier cohorts or if missing data are not random. Additionally, only limited supply-side variables were available and accessible. Travel time was then computed to all health facilities in Kenya because we could not distinguish those providing delivery or birth registration services. Using proxy government offices to geolocate CROs where exact addresses were unavailable may have introduced location inaccuracies. Lastly, the study assessed general determinants of birth registration without evaluating the timeliness of registration (within 6 months of birth), presenting an area for future research.

## Conclusion

Birth registration coverage among children under 3 years has improved, between 2014 and 2022; however, many counties are lagging behind others. The progress reflects successful initiatives by the government and philanthropic agencies. Our findings show that the Kenya CRS could leverage opportunities within the health sectors to strengthen birth registration. Continued sensitising efforts within communities and social media platforms could further enhance birth registration. Recent digitisation drives by the government, including introduction of digital birth certificates alongside Unique Personal Identifier to identify citizens from birth, offer promising developments. However, to maximise impact, national-level interventions should be complemented by strategies that address unique community-level and subnational-level barriers to ensure every region is included in the pursuit of achieving universal birth registration. Consequently, future studies should examine the local associations of birth registration and its determinants to illuminate context-specific needs. These findings may also be informative for other settings with similar CRVS structures, although local adaptation remains essential.

## Supplementary material

10.1136/bmjph-2025-003850 online supplemental file 1

## Data Availability

Data are available upon reasonable request.

## References

[1] Setel PW, Macfarlane SB, Szreter S (2007). A scandal of invisibility: making everyone count by counting everyone. The Lancet.

[2] Mills S, Lee JK, Rassekh BM (2019). Benefits of linking civil registration and vital statistics with identity management systems for measuring and achieving Sustainable Development Goal 3 indicators. J Health Popul Nutr.

[3] United Nations Goal 16 | department of economic and social affairs. https://sdgs.un.org/goals/goal16#targets_and_indicators.

[4] AbouZahr C, Mathenge G, Brøndsted Sejersen T, Macfarlane SB, AbouZahr C (2019). The Palgrave Handbook of Global Health Data Methods for Policy and Practice.

[5] Phillips DE, AbouZahr C, Lopez AD (2015). Are well functioning civil registration and vital statistics systems associated with better health outcomes?. The Lancet.

[6] Macharia PM, Giorgi E, Thuranira PN (2019). Sub national variation and inequalities in under-five mortality in Kenya since 1965. BMC Public Health.

[7] Phillips DE, Adair T, Lopez AD (2018). How useful are registered birth statistics for health and social policy? A global systematic assessment of the availability and quality of birth registration data. Popul Health Metr.

[8] Mills S, Abouzahr C, Kim J (2017). Civil registration and vital statistics for monitoring the sustainable development goals. https://hdl.handle.net/10986/27533.

[9] Krafft M Interoperability of crvs and eir systems for improved epi management in rwanda. https://dhis2.org/rwanda-crvs-eir-integration/.

[10] Rwanda launches new integrated civil registration system 2025. https://www.minaloc.gov.rw/news-detail/rwanda-launches-new-integrated-civil-registration-system.

[11] Cobos Muñoz D, de Savigny D, Sorchik R (2020). Better data for better outcomes: the importance of process mapping and management in CRVS systems. BMC Med.

[12] Mikkelsen L, Phillips DE, AbouZahr C (2015). A global assessment of civil registration and vital statistics systems: monitoring data quality and progress. The Lancet.

[13] Suthar AB, Khalifa A, Yin S (2019). Evaluation of approaches to strengthen civil registration and vital statistics systems: A systematic review and synthesis of policies in 25 countries. PLoS Med.

[14] Kumar K, Saikia N, Diamond-smith N (2022). Performance barriers of Civil Registration System in Bihar: An exploratory study. PLoS ONE.

[15] Yokobori Y, Obara H, Sugiura Y (2021). Gaps in the civil registration and vital statistics systems of low- and middle-income countries and the health sector’s role in improving the situation. *GHM*.

[16] Hassfurter K (2017). A snapshot of civil registration in sub-saharan africa. https://data.unicef.org/resources/snapshot-civil-registration-sub-saharan-africa/.

[17] Civil Registration Services (CRS) (2022). Kenya vital statistics report 2021.

[18] Civil Registration Services (CRS) (2022). Kenya vital statistics report.

[19] Civil Registration Services (2024). Kenya vital statistics report 2023.

[20] Bhatia A, Ferreira LZ, Barros AJD (2017). Who and where are the uncounted children? Inequalities in birth certificate coverage among children under five years in 94 countries using nationally representative household surveys. Int J Equity Health.

[21] Adair T, Badr A, Mikkelsen L (2023). Global analysis of birth statistics from civil registration and vital statistics systems. Bull World Health Org.

[22] Juma C, Beguy D, Mberu B (2016). Levels Of and Factors Associated with Birth Registration in the slums of Nairobi. *aps*.

[23] Pelowski M, Wamai RG, Wangombe J (2015). Why Don’t You Register Your Child? A Study of Attitudes and Factors Affecting Birth Registration in Kenya, and Policy Suggestions. J Dev Stud.

[24] Pelowski M, Wamai RG, Wangombe J (2016). How Would Children Register Their Own Births? Insights from a Survey of Students Regarding Birth Registration Knowledge and Policy Suggestions in Kenya. PLoS One.

[25] Wakibi S, Ngure E (2021). An Assessment of Knowledge, Attitude, and Practices of Birth and Death Registration in Kilifi County in the Coastal Region in Kenya. Biomed Res Int.

[26] Mathenge GW, Lehohla PJ, Makokha AO (2013). Factors associated with low levels of birth & death registration in Kieni East district of the Central Province of Kenya. Afr J Health Sci.

[27] Robert BN, Macharia PM, Lessani MN (2025). Spatially varying relationships between birth registration and influencing factors in Kenya, using a suite of geographically weighted regressions. Spat Spatiotemporal Epidemiol.

[28] County information | state department for devolution.

[29] CAP. http://kenyalaw.org:8181/exist/kenyalex/actview.xql?actid=CAP.%20149.

[30] Demographic and health survey (DHS). https://rspatialdata.github.io/dhs-data.html.

[31] The dhs program - dhs methodology. https://dhsprogram.com/Methodology/Survey-Types/DHS-Methodology.cfm#CP_JUMP_16156.

[32] CHS Guide to dhs statistics DHS-8. https://dhsprogram.com/data/Guide-to-DHS-Statistics/index.htm#t=Analyzing_DHS_Data.htm.

[33] KNBS and ICF (2023). Kenya demographic and health survey 2022.

[34] KNBS and ICF (2023). Kenya demographic and health survey 2022.

[35] (2023). Guide to dhs statistics.

[36] Gething P, Tatem A, Bird T (2015). Creating spatial interpolation surfaces with dhs data.

[37] Ngandu NK, Manda S, Besada D (2016). Does adjusting for recall in trend analysis affect coverage estimates for maternal and child health indicators? An analysis of DHS and MICS survey data. Glob Health Action.

[38] Duff P, Kusumaningrum S, Stark L (2016). Barriers to birth registration in Indonesia. Lancet Glob Health.

[39] Aboagye RG, Okyere J, Seidu A-A (2023). Determinants of birth registration in sub-Saharan Africa: evidence from demographic and health surveys. Front Public Health.

[40] Wodon Q, Yedan A (2019). Obstacles to birth registration in Niger: estimates from a recent household survey. J Health Popul Nutr.

[41] Jackson M, Duff P, Kusumaningrum S (2014). Thriving beyond survival: understanding utilization of perinatal health services as predictors of birth registration: a cross-sectional study. BMC Int Health Hum Rights.

[42] (2005). The “rights” start to life: a statistical analysis of birth registration.

[43] Isara Socio-demographic determinants of birth registration among mothers in an urban community in southern nigeria. https://jmedtropics.org/article.asp?issn=2276-7096;year=2015;volume=17;issue=1;spage=16;epage=21;aulast=Isara.

[44] Fagernäs S, Odame J (2013). Birth registration and access to health care: an assessment of Ghana’s campaign success. Bull World Health Organ.

[45] Amo-Adjei J, Annim SK (2015). Socioeconomic determinants of birth registration in Ghana. BMC Int Health Hum Rights.

[46] Sharma SK, Ghimire DR, Adhikari D (2023). Birth registration in Nepal: An assessment of progress based on two national surveys. *PLOS Glob Public Health*.

[47] Duryea S, Olgiati A, Stone L (2006). The Under-Registration of Births in Latin America. SSRN Journal.

[48] Makinde O, Olapeju B, Ogbuoji O (2016). Trends in the completeness of birth registration in Nigeria: 2002-2010. DemRes.

[49] Tang W, Zou L (2022). Trends and characteristics of multiple births in Baoan Shenzhen: A retrospective study over a decade. Front Public Health.

[50] Anaduaka US (2022). Multilevel analysis of individual- and community-level determinants of birth certification of children under-5 years in Nigeria: evidence from a household survey. BMC Public Health.

[51] Kumar K, Saikia N (2021). Determinants of birth registration in India: Evidence from NFHS 2015–16. PLoS ONE.

[52] Pont AV, Hafid F, Ramadhan K (2023). Factors associated with birth registrations in Indonesia. *ELECTRON J GEN MED*.

[53] Adi AE, Abdu T, Khan A (2015). Understanding whose births get registered: a cross sectional study in Bauchi and Cross River states, Nigeria. BMC Res Notes.

[54] (2019). What is birth registration and why does it matter. https://www.unicef.org/stories/what-birth-registration-and-why-does-it-matter.

[55] Balogun OO, K C Bhandari A, Tomo CK (2023). Association of sociodemographic and maternal healthcare factors with birth registration in Angola. Public Health (Fairfax).

[56] Xu F, Sullivan EA, Black DA (2012). Under-reporting of birth registrations in New South Wales, Australia. BMC Pregnancy Childbirth.

[57] Abay ST, Gebre-Egziabher AG (2020). Status and associated factors of birth registration in selected districts of Tigray region, Ethiopia. BMC Int Health Hum Rights.

[58] Arora A Birth registration for every child by 2030: are we on track? unicef data 2019. https://data.unicef.org/resources/birth-registration-for-every-child-by-2030/.

[59] Wendt A, Hellwig F, Saad GE (2022). Birth registration coverage according to the sex of the head of household: an analysis of national surveys from 93 low- and middle-income countries. BMC Public Health.

[60] Nomura M, Xangsayarath P, Takahashi K (2018). Socioeconomic determinants of accessibility to birth registration in Lao PDR. BMC Public Health.

[61] Solanke BL, Oladosu OA, Akinlo A (2015). Religion as a Social Determinant of Maternal Health Care Service Utilisation in Nigeria. *APS*.

[62] Bequele A (2005). Universal birth registration: the challenge in africa.

[63] Schiavina M, Melchiorri M, Pesaresi M (2023). GHS-smod r2023a - ghs settlement layers, application of the degree of urbanisation methodology (stage i) to ghs-pop r2023a and ghs-built-s r2023a.

[64] European Commission (2021). Applying the degree of urbanisation: a methodological manual to define cities, towns and rural areas for international comparisons: 2021 edition.

[65] Ray N, Ebener S (2008). AccessMod 3.0: computing geographic coverage and accessibility to health care services using anisotropic movement of patients. Int J Health Geogr.

[66] Moturi AK, Suiyanka L, Mumo E (2022). Geographic accessibility to public and private health facilities in Kenya in 2021: An updated geocoded inventory and spatial analysis. Front Public Health.

[67] (2023). Kenya demographic and health survey 2022 (pr143). key indicators report.

[68] Gollini I, Lu B, Charlton M (2015). GWmodel: An R Package for Exploring Spatial Heterogeneity Using Geographically Weighted Models. J Stat Soft.

[69] Agency KN (2023). Over 3800 people registered in mobile outreach. https://www.kenyanews.go.ke/over-3800-people-registered-in-mobile-outreach/.

[70] UNHCR Birth certificates signal brighter future for stateless children in kenya. https://www.unhcr.org/news/stories/birth-certificates-signal-brighter-future-stateless-children-kenya.

[71] EastAfrican End of statelessness in sight for shona as kenya issues birth certificates - the eastafrican 2020. https://www.theeastafrican.co.ke/tea/news/east-africa/end-of-statelessness-in-sight-for-shona-as-kenya-issues-birth-certificates-1428956.

[72] Plan International Kenya (2020). Investment case for birth registration in kenya.

[73] (2023). Newborn and child health stategic plan 2022-2026.

[74] Measure Evaluation (2013). National civil registration and vital statistics system baseline systems assessment report.

[75] The Star PS bitok: civil registration centers will be in all constituencies in three year. https://www.the-star.co.ke/news/realtime/2024-06-04-ps-bitok-civil-registration-centers-will-be-in-all-constituencies-in-three-years.

[76] (2025). Birth registration in sub-saharan africa: current levels and trends. https://data.unicef.org/resources/birth-registration-in-sub-saharan-africa-current-levels-and-trends/.

[77] Kakamega civil society leaders launch new alliance to improve health for women, newborns, and children. https://www.path.org/our-impact/media-center/kakamega-civil-society-leaders-launch-new-alliance-to-improve-health-for-women-newborns-and-children/.

[78] Tutunze Kakamega (2024). Experiences and Lessons from SDR Implementation in Kakamega County.

[79] Measure Evaluation PIMA (2018). Strengthening Civil Registration and Vital Statistics in Kenya.

[80] County launched a 30- day rapid results initiative – kenya news agency 2019. https://www.kenyanews.go.ke/county-launched-a-30-day-rapid-results-initiative/.

[81] Parliament of Kenya Implementation of house resolution on establishment of civil registration centers on course, state department affirms | parliament of kenya 2024. http://217.21.116.45/parliament/node/1167.

[82] (2020). What is the true human toll of covid-19? for better answers to this critical question, strengthen civil registration systems. https://www.vitalstrategies.org/what-is-the-true-human-toll-of-covid-19-for-better-answers-to-this-critical-question-strengthen-civil-registration-systems/.

[83] Kelly M, Mathenge G, Rao C (2021). Lessons Learnt and Pathways forward for National Civil Registration and Vital Statistics Systems after the COVID-19 Pandemic. JEGH.

[84] AbouZahr C, Bratschi MW, Cercone E (2021). The COVID-19 Pandemic: Effects on Civil Registration of Births and Deaths and on Availability and Utility of Vital Events Data. Am J Public Health.

[85] WHO Improving civil registration, vital statistics and health data through strengthened partnerships in kenya 2022. https://www.who.int/news-room/feature-stories/detail/strengthening-health-data-kenya.

[86] (2023). The Civil Registration Vital Statistics and Identity (CRVSID) Country Case Studies -KENYA.

[87] Ebbers AL, Smits J (2022). Household and context-level determinants of birth registration in Sub-Saharan Africa. PLoS One.

[88] Government of kenya (GOK) Ecitizen.go.ke - government of kenya services simplified. https://www.ecitizen.go.ke/.

[89] KNBS (2019). Kenya population and housing census reports. https://www.knbs.or.ke/2019-kenya-population-and-housing-census-reports/.

[90] MP wants civil registration offices decentralised for easy access. https://www.the-star.co.ke/counties/north-eastern/2023-03-19-mp-wants-civil-registration-offices-decentralised-for-easy-access.

[91] (2020). Kenya abolishes punitive citizenry policies targeting somalis. https://www.garoweonline.com/en/world/africa/kenya-abolishes-punitive-citizenry-policies-targeting-somalis.

[92] Huduma kenya. https://www.hudumakenya.go.ke/lifeEvent/ServiceDetails/14.

[93] Kenya law: birth registration in perspective - J. E. O. https://kenyalaw.org/kl/index.php?id=1897.

[94] Citizen Digital News Kenya registers increased births by single mothers 2023. https://www.citizen.digital/news/kenya-registers-increased-births-by-single-mothers-n319161.

[95] Plan International Kenya (2020). Handbook for community led birth registration in kenya.

[96] Corbacho A, Osorio Rivas R (2012). Travelling the distance: a gps-based study of the access to birth registration services in latin america and the caribbean.

[97] Rutstein SO, Johnson K (2004). The dhs wealth index.

