# Supplementary material for: Missed at birth: a cross-sectional analysis of national determinants and subnational changes in birth registration coverage in Kenya, using 2014 and 2022 Demographic and Health Survey (DHS) data
Source: BMJ Public Health. 2026 Apr 13;4(2):e003850. doi: 10.1136/bmjph-2025-003850 (PMC13084874; doi:10.1136/bmjph-2025-003850)
Supplement: online supplemental file 1 [file bmjph-4-2-s001.docx]

**Supplementary Files**

**
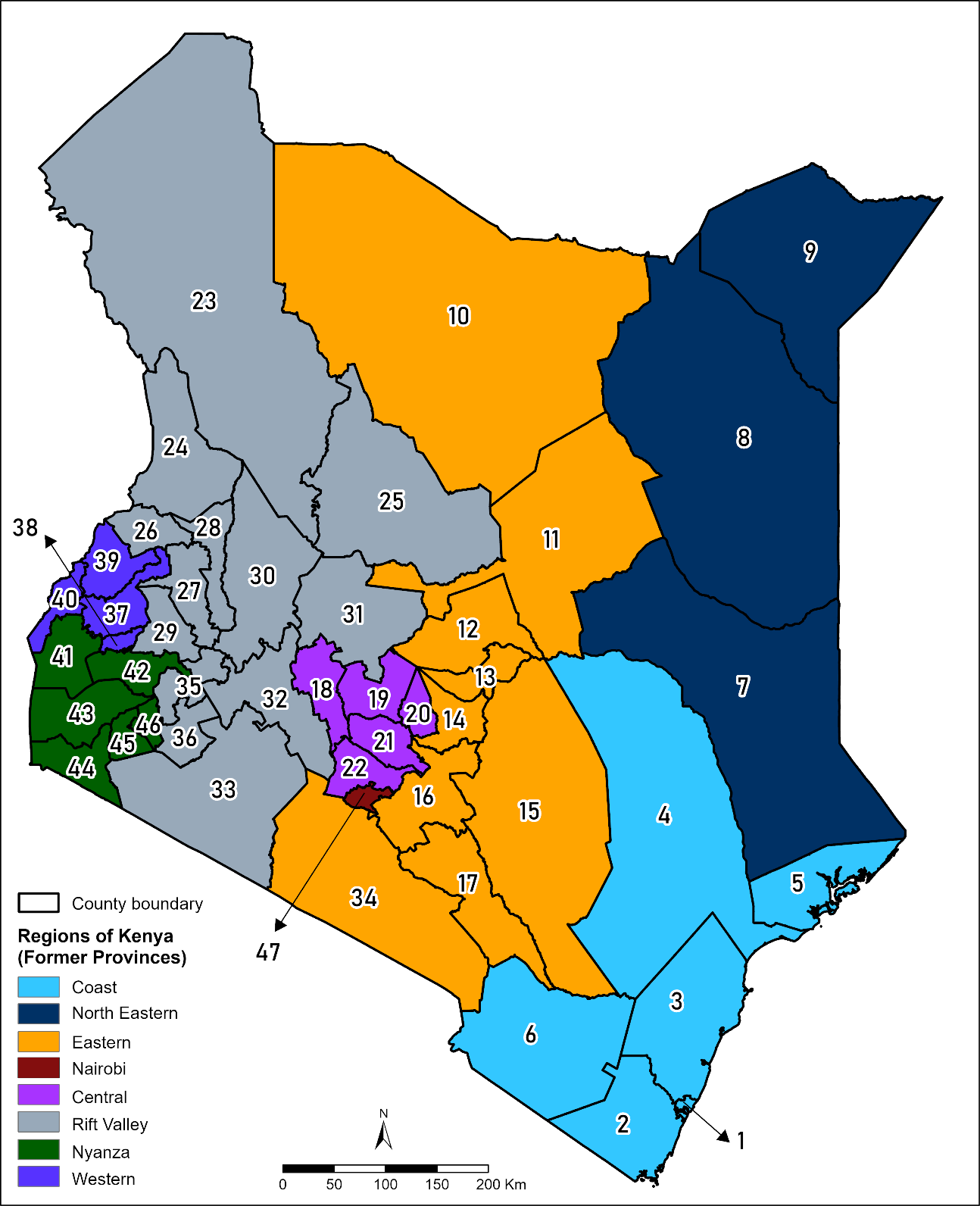
**

Figure S1. Map showing the 47 counties of Kenya and former 8 provinces.

*Mombasa [1], Kwale [2], Kilifi [3], Tana River [4], Lamu [5], Taita Taveta [6], Garissa[7], Wajir [8], Mandera [9], Marsabit [10], Isiolo [11], Meru [12], Tharaka-Nithi [13], Embu [14], Kitui [15], Machakos [16], Makueni [17], Nyandarua [18], Nyeri [19], Kirinyaga [20], Murang’a [21], Kiambu [22], Turkana [23], West Pokot [24], Samburu [25], Trans Nzoia [26], Uasin Gishu [27], Elgeyo-Marakwet [28], Nandi [29], Baringo[30],Laikipia [31], Nakuru [32], Narok [33], Kajiado [34],Kericho[35], Bomet [36], Kakamega [37], Vihiga [38], Bungoma[39], Busia [40], Siaya [41], Kisumu [42], Homa Bay [43], Migori [44], Kisii [45], Nyamira [46], Nairobi [47].*

Table S1. A summary of birth registration questions asked for children under 5 years and their response options within the Demographic and Health Survey (DHS) for the years 2014 and 2022.

| **DHS survey year** | **Recode** | **Birth Registration question** | **Response options** |
| --- | --- | --- | --- |
| **2014** | **DHS-VI** | Has (NAME) ever been registered with the civil authority? | 1=Yes registered, with a birth certificate  2=Yes registered without birth certificate  8=Don’t Know  3=Not Registered |
| **2022** | **DHS-VII** | Does (FIRST NAME) have a birth certificate? IF NO, PROBE: Has (FIRST NAME)'s birth ever been registered with the civil authority? | 1= Has Certificate  2=Registered  3=Neither  8=Don’t know |

**An overview of birth registration process in Kenya.**

**
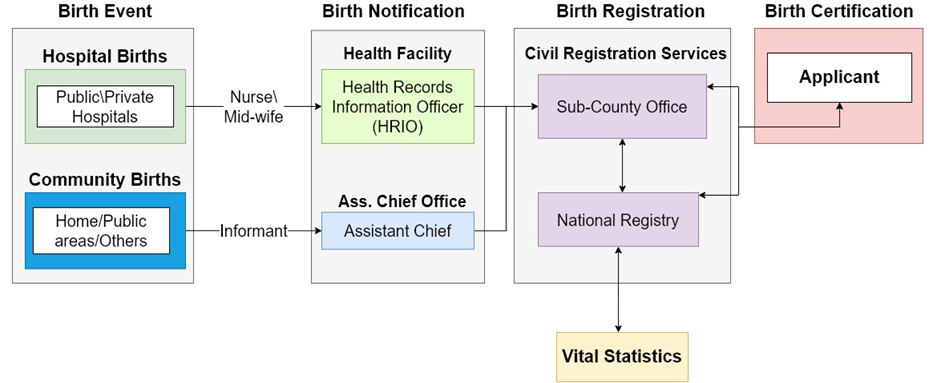
**

Figure S2. An overview of birth registration process in Kenya. (Currently Kenyan Citizens in some parts of the country can apply for birth certificates through Huduma centres (physical one-stop-shop service delivery point for government services) and online through E-Citizen platform (digital portal that allows citizens to access government services).

**Determinants of birth registration**


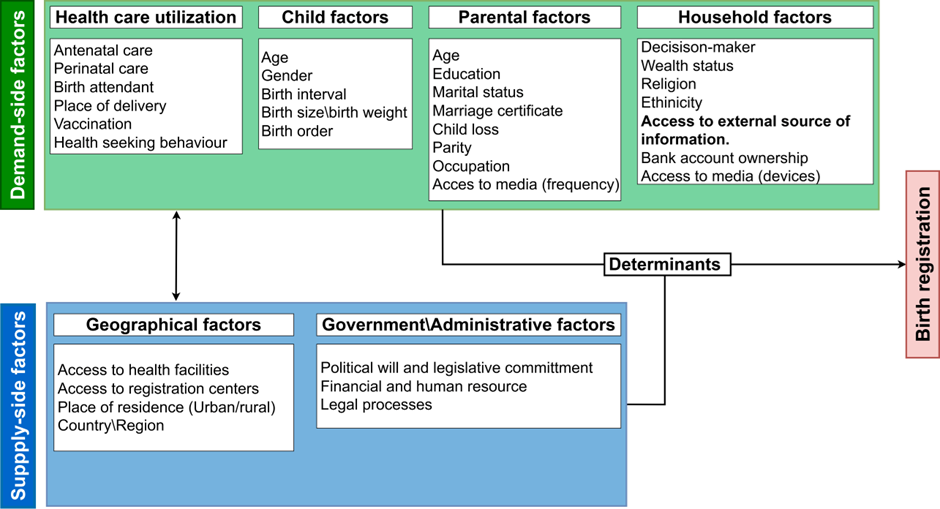


Figure S3. Conceptual framework of determinants of birth registration based on literature review

Table S2. A summary of determinants of birth registration and rationale as documented in literature.

| **Category** | **Determinants of Birth Registration** | **Rationale** |
| --- | --- | --- |
| **Health care utilization** | Antenatal care. | Contact with the formal health system or health personnel increases the chances of receiving information about the needs and process of birth registration and certification [1,2]. It also includes direct provision of (birth notification card), which is the first requisite document required to complete the birth registration process. Further, women who deliver by traditional birth attendants or at home need to travel to a clinic for a notification, creating an additional barrier to birth registration [1]. |
|  | Antenatal care visits |  |
|  | Postnatal care. |  |
|  | Skilled-birth attendant  (by health personnel) |  |
|  | Health seeking behavior (e.g., Fever\cough treatment,  Diarrhea treatment) |  |
|  | Place of delivery (health facility) | Delivery in health facilities is shown to improve birth registration [3] compared to home deliveries. For instance, in government hospitals, children tend to be registered immediately after birth [2]. |
|  | Vitamin A supplementation | Improving access to healthcare, including vitamin A supplementation, promotes interaction with health care providers and can increase the likelihood of parents registering their child's birth [4]. |
|  | Vaccination | Immunization provides an opportunity to interact with health care workers, such that the absence of a health card or birth certificate can serve as a point of entry for child’s registration [2]. |
| **Child factors** | Age | The likelihood of birth registration varies with the age of the child [5]. Though some studies show relationship between birth registration and child age is nonlinear [6], older children approaching age 5 are most likely to have birth certificate as they approach school enrollment age where a birth certificate is required for school admission [7,8]. |
|  | Gender | Gender may influence a child’s birth registration especially in patriarchal societies. In such cultures girls may be discriminated against and viewed as less valuable than boys, leading to parents neglecting or failing to prioritize registration of their daughters [7,9]. |
|  | Birth interval (Length of time between two successive live births) | Women who have experienced a short birth interval are prone to several health problems including, antepartum hemorrhage, anemia, premature rupture of membrane, and increased risk of infant mortality rate, preterm delivery, low birth weights among others [10]. Such complications can delay birth registration process or result in non-registration [11]. |
|  | Size-at-birth used proxy for child’s birth where the quality of weight data is poor | Childbirth weight can be used as an indicator of parental investment in child development [11]. |
|  | Birth order | Lack of immediate benefits from registering the first child may affect the likelihood of later-born children being registered. Further, resources tend to be depleted caring for larger families, thus making it costly to commit to registering and certifying younger children [11]. |
| **Parental factors** | Maternal age | Birth registration varies by the age of mother at the time of the child’s birth [8,11]. Children born to non-adolescent mothers (>20 years) have higher odds of birth certification, which declines after 35 years. This could be explained by time demand in and out of home for mothers at this age, which can lead them to postpone certification of their child [11]. Younger mother are also less aware of the registration process and childcare experience [12]. |
|  | Paternal age | One study shows the proportion of children registered increase with father’s age before decreasing after 44 years. The association with birth registration was not significant [11]. |
|  | Maternal education | Education status has a great influence of the level of awareness of many factors, including importance of birth registration [3,13–15] and health care utilization associated with improved birth registration [11]. |
|  | Paternal education |  |
|  | Maternal marital status | Married women are more likely to have their children registered than unmarried women [16]. Married women could have better knowledge and birth registration practices compared to unmarried or single women [3]. This could be explained by the sharing of information related to childcare with a spouse who could have a higher education, hence better awareness of birth registration. Additionally married women are more likely to receive support from partner such as cost in accessing registration centers [13]. |
|  | Marriage certificate ownership | In some countries, a marriage certificate is a requisite document for birth registration [14,17]. Lack of which may hinder birth registration process. Further, in some societies, mothers may face gender discrimination while registering their child if they lack an ID or marriage certificate [18]. |
|  | Child loss | The loss of a child can cause trauma. As a result, the parent may elect to delay or not register the birth of the deceased child [11]. |
|  | Parity (number of live births a woman has had during her lifetime) | Mother having more pregnancies is associated with non-registration [19–21]*.* Women with multiple births are more likely to experience adverse health outcome both on the mother and the child, thus diverting attention from administrative duties such as birth registration [10] . Additionally, those from poor families may face financial burden impacting their ability to register their children [22]. |
|  | Decision-maker (Female) | Female-headed households may lack the necessary economic resources to facilitate access and affordability of registering their child’s birth. In some societies fathers may be considered as the primarily responsible individuals for birth registration, leading to additional bureaucratic challenges or confusion for households headed by females in the absence of a male parent [13]. On the other hand empowered women who are heading households have greater bargaining power in the family, thus prioritizing allocation of resources to their children [23]. |
|  | Occupation | Household heads working in the formal public sector are more likely to be familiar with the importance and process of birth registration, hence the higher likelihood of registering their children compared to those working in the informal sectors [24]. |
| **Household factors** | Wealth status | A child belonging to a middle-income or rich household has a higher likelihood of birth certification. Richer households may be more aware of the importance of a birth certificate for a child’s future due to exposure such as university education, traveling abroad. They are also in a better position to afford the costs associated with the registration process [11,6,25,26]. |
|  | Religion | Religious beliefs or practices could influence women’s reproductive health practices, including the utilization of health care services such as skilled birth attendants, antenatal visits, skilled birth attendants etc., minimizing their chances of interacting with the formal health system which would raise awareness on birth registration [27]. Some religions such as Orthodox Christianity require children to be baptized to be counted as members. A baptism card is issued, and some parents may consider this an official birth certificate, abandoning the official registration of their children [28]. |
|  | Ethnicity | Ethnicity may be associated with certain religions and cultural practices that influence health practices such as the type of care sought (traditional healer as opposed to doctor). A study in Nigeria has shown children belonging to certain ethnic groups (Yoruba) had higher chances of being certified compared to others (Hausa/Fulani) [11,25,26]. |
| **Access to external Source of Communication** | Bank account ownership | Parents or caregivers who have made contact with and interacted with the formal financial system may be knowledgeable about the importance of a birth certificate, e.g., a birth certificate can serve as proof of age when opening a bank account [11]. |
|  | Access to media | Access to social media platforms such as TV, radio, smart phones is a good channel to raise awareness about birth registration including institutions responsible for the registration process. However, the effectiveness of mass media depends on mode of transmission and/or how people understand the information [3]. |
| **Geographical factors** | Access to health facilities | The greater the distance to the registration centers the higher the financial and opportunity costs for the family. Poor families are less likely to incur transportation cost to a registration center [17]. |
|  | Access to registration centers |  |
|  | Place of residence (Urban or Rural) | People living in rural areas are faced with increased financial and opportunity cost such as increased transportation costs due to a lack of poor infrastructure including roads and health facilities [17]. |
|  | County/Region | Regional disparities in resources, such as to health facilities and targeting interventions and policies in specific regions are some of the factors that would either positively or negatively impact birth registration [7]. Further, significant spatial variation in child registration and could possibly be explained by the presence of local initiatives such as promotion of maternal and child health in some regions, contributing to the spatial differences in birth registration [6]. |
| **Government/**  **Administrative** | Political will and legislative commitment | The extent to which the government or key decision-makers are willing to prioritize birth registration including raising awareness about birth registration, investing in infrastructures such as hospitals and birth registration centers, could contribute to low or increased birth registration rates [6,28]. |
|  | Inadequate financial and human resource | Lack of adequate and relevant manpower may cause delays in the registration process, discouraging people from registering their children. Constrained financial resources may limit expansion of infrastructure and public awareness campaigns to promote birth registration [28,29]. |
|  | Legal issues | Legal requirements for procedures for late may require the services of a lawyer and can be frustrating and costly, delaying or hindering birth registration [8]. |

**
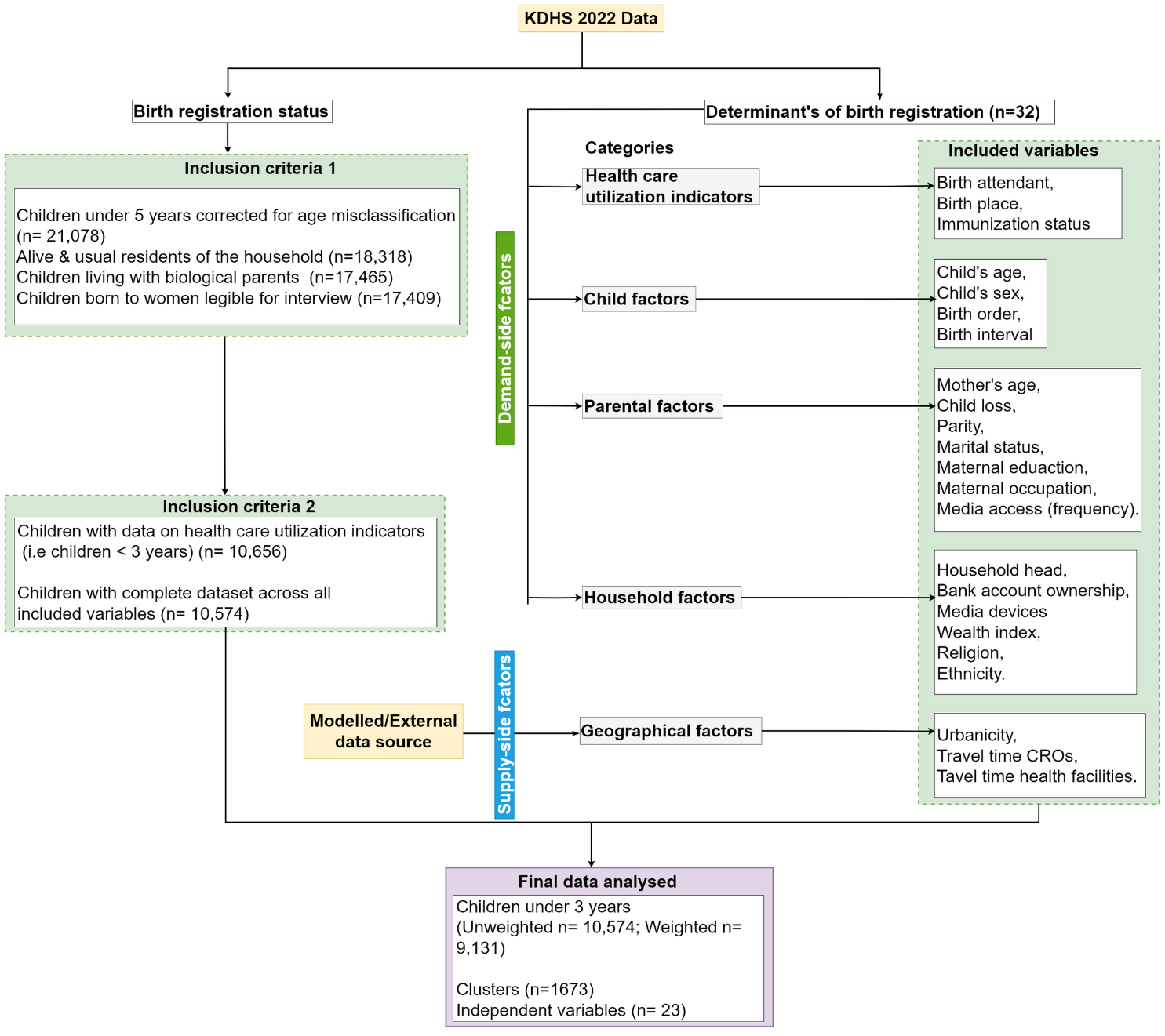
**

Figure S4. Summary of determinants data extraction process from DHS 2022 survey and external geospatial covariates.

**Geographical access to Civil Registration Offices (CROs).**

The accessibility model was implemented using the AccessMod 5 tool, employing the least cost path algorithm to compute the fastest route between a geographical point and a point of interest (i.e., CROs) [30]. A list of CROs in Kenya and their addresses, was obtained from the Kenya Civil Registration Services (CRS). The CROs were then geolocated using Google Earth/Maps and digital place-name gazetteers, including Geonames and OpenStreetMap. In instances where exact addresses were unavailable, coordinates for government offices, such as Deputy County Commissioners’ (DCC) offices, were used to locate CROs, as advised by the CRS. This approach was sensible, given that most CROs are within the DCC's sub-county headquarters. The points were validated through mapping to confirm their alignment with the designated sub-counties and counties.

An optimistic travel scenario intended to reflect the travel behaviors of most of the Kenyan population was utilized [31–35]. It is assumed that individuals first walk to the nearest road and select an available mode of transportation (bicycle, motorcycle, or vehicle) based on the terrain and infrastructure present. The accessibility model was adjusted for various landscape elements that either facilitate or hinder travel, including land use, elevation, road types, travel speeds, water bodies, and protected areas. Transport barriers, such as major rivers, water bodies, and forests, were considered impassable and assigned a speed of zero. Essential spatial data for modeling travel times and their sources are outlined in Table S3. The travel speeds for differing land covers and road categories were sourced from prior accessibility research in Kenya [32,35], as detailed in Table S4. Additionally, the slope obtained from the Digital Elevation Model (DEM) was employed to factor in how topography affects walking and biking speeds [36]. The resultant raster serves as a cost friction surface from which travel time was derived, is illustrated Figure S3.

Table S3. Summary of ancillary data used for modelling access to civil registration offices.

| **Ancillary datasets** | **Spatial Resolution** | **Source** |
| --- | --- | --- |
| Road network (classified as primary, secondary, county, and rural roads) | - | Kenya Roads Board [37] and Open Street Map [38] |
| Landcover | 10m x 10m | ESRI Land Cover [39] |
| Digital Elevation Model (DEM) | 30m × 30m | Shuttle Radar Topographic Mission (SRTM) at RCMRD Geoportal [40]. |
| Water body (e.g. Lakes, major rivers) | - | Global lakes and wetlands database [41] |
| Protected areas (e.g. forests, parks) | - | Protected Planet [42] |

Table S4. Travel speed adopted across various land cover classes.

| **Land cover category** | **Speed (Km/Hr)** | **Mode of transport** |
| --- | --- | --- |
| Primary roads | 50.00 | Motorized |
| Secondary roads | 30.00 | Motorized |
| County roads | 10.00 | Bicycling |
| Rural roads | 5.00 | Walking |
| Tree Cover | 2.50 | Walking |
| Flooded vegetation | 0.10 | Walking |
| Cropland | 4.00 | Walking |
| Built-Up | 5.00 | Walking |
| Bare ground | 5.00 | Walking |
| Waterbodies | 0.00 | Walking |

**
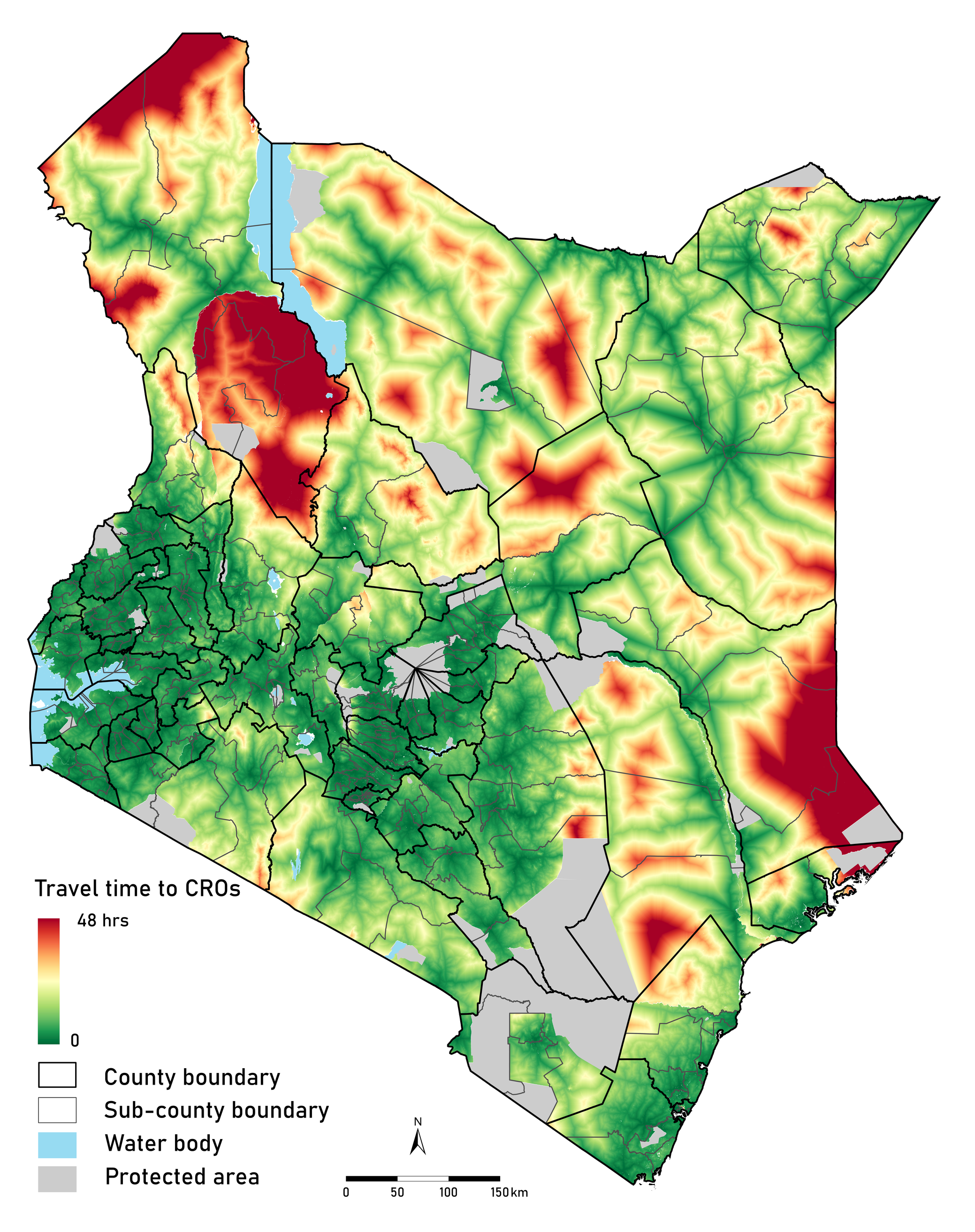
**

Figure S5. Travel time to Civil Registration Offices in hours.

**References**

1 Jackson M, Duff P, Kusumaningrum S, *et al.* Thriving beyond survival: Understanding utilization of perinatal health services as predictors of birth registration: A cross-sectional study. *BMC International Health and Human Rights*. 2014;14:306. doi: 10.1186/s12914-014-0038-3

2 UNICEF. *The ‘rights’ Start to Life: A Statistical Analysis of Birth Registration*. UNICEF 2005.

3 Isara. Socio-demographic determinants of birth registration among mothers in an urban community in southern Nigeria. https://jmedtropics.org/article.asp?issn=2276-7096;year=2015;volume=17;issue=1;spage=16;epage=21;aulast=Isara (accessed 6 March 2023)

4 Fagernäs S, Odame J. Birth registration and access to health care: an assessment of Ghana’s campaign success. *Bull World Health Organ*. 2013;91:459–64. doi: 10.2471/BLT.12.111351

5 Mathenge GW, Lehohla PJ, Makokha AO, *et al.* Factors associated with low levels of birth & death registration in Kieni East district of the Central Province of Kenya. *African Journal of Health Sciences*. 2013;26:272–90. doi: 10.4314/ajhs.v26i4

6 Amo-Adjei J, Annim SK. Socioeconomic determinants of birth registration in Ghana. *BMC Int Health Hum Rights*. 2015;15:14. doi: 10.1186/s12914-015-0053-z

7 Sharma SK, Ghimire DR, Adhikari D, *et al.* Birth registration in Nepal: An assessment of progress based on two national surveys. *PLOS Global Public Health*. 2023;3:e0000759. doi: 10.1371/journal.pgph.0000759

8 Duryea S, Olgiati A, Stone L. The Under-Registration of Births in Latin America. 2006.

9 Makinde OA, Olapeju B, Ogbuoji O, *et al.* Trends in the completeness of birth registration in Nigeria: 2002–2010. *Demographic Research*. 2016;35:315–38.

10 Tang W, Zou L. Trends and characteristics of multiple births in Baoan Shenzhen: A retrospective study over a decade. *Front Public Health*. 2022;10. doi: 10.3389/fpubh.2022.1025867

11 Anaduaka US. Multilevel analysis of individual- and community-level determinants of birth certification of children under-5 years in Nigeria: evidence from a household survey. *BMC Public Health*. 2022;22:2340. doi: 10.1186/s12889-022-14786-2

12 Kumar K, Saikia N. Determinants of birth registration in India: Evidence from NFHS 2015-16. *PLoS One*. 2021;16:e0257014. doi: 10.1371/journal.pone.0257014

13 Aboagye RG, Okyere J, Seidu A-A, *et al.* Determinants of birth registration in sub-Saharan Africa: evidence from demographic and health surveys. *Front Public Health*. 2023;11:1193816. doi: 10.3389/fpubh.2023.1193816

14 Pont AV, Hafid F, Ramadhan K, *et al.* Factors associated with birth registrations in Indonesia. *ELECTRON J GEN MED*. 2023;20:em462. doi: 10.29333/ejgm/12900

15 Sharma SK, Ghimire DR, Adhikari D, *et al.* Birth registration in Nepal: An assessment of progress based on two national surveys. *PLOS Glob Public Health*. 2023;3:e0000759. doi: 10.1371/journal.pgph.0000759

16 Adi AE, Abdu T, Khan A, *et al.* Understanding whose births get registered: a cross sectional study in Bauchi and Cross River states, Nigeria. *BMC Res Notes*. 2015;8:79. doi: 10.1186/s13104-015-1026-y

17 Duff P, Kusumaningrum S, Stark L. Barriers to birth registration in Indonesia. *The Lancet Global Health*. 2016;4:e234–5. doi: 10.1016/S2214-109X(15)00321-6

18 UNICEF. What is birth registration and why does it matter? 2019. https://www.unicef.org/stories/what-birth-registration-and-why-does-it-matter (accessed 7 March 2023)

19 Balogun OO, K C Bhandari A, Tomo CK, *et al.* Association of sociodemographic and maternal healthcare factors with birth registration in Angola. *Public Health*. 2023;223:94–101. doi: 10.1016/j.puhe.2023.07.026

20 Xu F, Sullivan EA, Black DA, *et al.* Under-reporting of birth registrations in New South Wales, Australia. *BMC Pregnancy Childbirth*. 2012;12:147. doi: 10.1186/1471-2393-12-147

21 Abay ST, Gebre-Egziabher AG. Status and associated factors of birth registration in selected districts of Tigray region, Ethiopia. *BMC Int Health Hum Rights*. 2020;20:20. doi: 10.1186/s12914-020-00235-x

22 Arora A. Birth Registration for Every Child by 2030: Are we on track? UNICEF DATA. 2019. https://data.unicef.org/resources/birth-registration-for-every-child-by-2030/ (accessed 3 February 2024)

23 Wendt A, Hellwig F, Saad GE, *et al.* Birth registration coverage according to the sex of the head of household: an analysis of national surveys from 93 low- and middle-income countries. *BMC Public Health*. 2022;22:1942. doi: 10.1186/s12889-022-14325-z

24 Wodon Q, Yedan A. Obstacles to birth registration in Niger: estimates from a recent household survey. *J Health Popul Nutr*. 2019;38:26. doi: 10.1186/s41043-019-0185-1

25 Juma C, Beguy D, Mberu B. Levels Of and Factors Associated with Birth Registration in the slums of Nairobi. *African Population Studies*. 2016;30. doi: 10.11564/30-2-848

26 Nomura M, Xangsayarath P, Takahashi K, *et al.* Socioeconomic determinants of accessibility to birth registration in Lao PDR. *BMC Public Health*. 2018;18:116. doi: 10.1186/s12889-017-5009-x

27 Solanke BL, Oladosu OA, Akinlo A, *et al.* Religion as a Social Determinant of Maternal Health Care Service Utilisation in Nigeria. *African Population Studies*. 2015;29. doi: 10.11564/29-2-761

28 Abay ST, Gebre-egziabher AG. Status and associated factors of birth registration in selected districts of Tigray region, Ethiopia. *BMC International Health and Human Rights*. 2020;20:20. doi: 10.1186/s12914-020-00235-x

29 Bequele A. Universal birth registration: The challenge in Africa. 2005.

30 Ray N, Ebener S. AccessMod 3.0: computing geographic coverage and accessibility to health care services using anisotropic movement of patients. *Int J Health Geogr*. 2008;7:63. doi: 10.1186/1476-072X-7-63

31 Joseph NK, Macharia PM, Ouma PO, *et al.* Spatial access inequities and childhood immunisation uptake in Kenya. *BMC Public Health*. 2020;20:1407. doi: 10.1186/s12889-020-09486-8

32 Macharia PM, Mumo E, Okiro EA. Modelling geographical accessibility to urban centres in Kenya in 2019. *PLOS ONE*. 2021;16:e0251624. doi: 10.1371/journal.pone.0251624

33 Mumo E, Agutu NO, Moturi AK, *et al.* Geographic accessibility and hospital competition for emergency blood transfusion services in Bungoma, Western Kenya. *International Journal of Health Geographics*. 2023;22:6. doi: 10.1186/s12942-023-00327-6

34 Ocholla IA, Agutu NO, Ouma PO, *et al.* Geographical accessibility in assessing bypassing behaviour for inpatient neonatal care, Bungoma County-Kenya. *BMC Pregnancy Childbirth*. 2020;20:287. doi: 10.1186/s12884-020-02977-x

35 Ouma PO, Malla L, Wachira BW, *et al.* Geospatial mapping of timely access to inpatient neonatal care and its relationship to neonatal mortality in Kenya. *PLOS Global Public Health*. 2022;2:e0000216. doi: 10.1371/journal.pgph.0000216

36 Joseph NK, Macharia PM, Ouma PO, *et al.* Spatial access inequities and childhood immunisation uptake in Kenya. *BMC Public Health*. 2020;20:1407. doi: 10.1186/s12889-020-09486-8

37 KRB. KENYA ROADS BOARD (KRB) MAP PORTAL. https://maps.krb.go.ke/kenya-roads-board12769/maps (accessed 1 March 2024)

38 OSM. Kenya Roads (OpenStreetMap Export) - Humanitarian Data Exchange. https://data.humdata.org/dataset/hotosm_ken_roads? (accessed 1 March 2024)

39 Esri. Esri Land Cover. https://livingatlas.arcgis.com/landcover (accessed 1 March 2024)

40 RCMRD. Kenya SRTM DEM 30meters. https://gmesgeoportal.rcmrd.org/datasets/rcmrd::kenya-srtm-dem-30meters/about (accessed 1 March 2024)

41 GLWD. Global Lakes and Wetlands Database - waterdata. https://wbwaterdata.org/dataset/global-lakes-and-wetlands-database (accessed 1 March 2024)

42 Explore the World’s Protected Areas. https://www.protectedplanet.net/en (accessed 1 March 2024)
